# Supplementary material for: 4-Hydroxy-2-nonenal antimicrobial toxicity is neutralized by an intracellular pathogen
Source: eLife. 2021 May 6;10:e59295. doi: 10.7554/eLife.59295 (PMC8174450; doi:10.7554/eLife.59295)
Supplement: Source data 1. [file elife-59295-data1.docx]

| Data presented here are sorted by q-value (statistical significance) < 0.05 | |  |  |  |  |  |
| --- | --- | --- | --- | --- | --- | --- |
| Synonym | Product | Expression mock | Expression HNE | qValue mock vs HNE | HNE fold over mock | lmo numbers |
| LMRG_00296 | oxidoreductase | 3 | 716 | 0 | 238.6666667 | lmo0613 |
| LMRG_00295 | hypothetical protein | 3 | 542 | 0 | 180.6666667 | lmo0612 |
| predicted RNA | antisense: LMRG_00484 | 15 | 2673 | 0 | 178.2 | #N/A |
| LMRG_00999 | mercuric ion binding protein | 2 | 332 | 0 | 166 | lmo1852 |
| LMRG_01869 | hypothetical protein | 41 | 6735 | 0 | 164.2682927 | lmo2829 |
| LMRG_02304 | peptidoglycan bound protein | 1 | 137 | 0 | 137 | lmo0880 |
| predicted RNA | - | 19 | 2296 | 0 | 120.8421053 | #N/A |
| LMRG_00177 | hypothetical protein | 63 | 6244 | 0 | 99.11111111 | lmo0496 |
| LMRG_02097 | ATP-dependent Clp protease ATP-binding subunit ClpE | 47 | 4494 | 0 | 95.61702128 | lmo0997 |
| LMRG_01000 | Cu2+-exporting ATPase | 33 | 2999 | 0 | 90.87878788 | lmo1853 |
| LMRG_01001 | hypothetical protein | 18 | 1628 | 0 | 90.44444444 | lmo1854 |
| LMRG_02351 | hypothetical protein | 3 | 260 | 0 | 86.66666667 | lmo0102 |
| LMRG_00484 | YceI like family protein | 75 | 5695 | 0 | 75.93333333 | lmo0796 |
| LMRG_01602 | hypothetical protein | 30 | 1977 | 0 | 65.9 | lmo2230 |
| predicted RNA | antisense: LMRG_02097 | 8 | 527 | 0 | 65.875 | #N/A |
| LMRG_02352 | hypothetical protein | 2 | 123 | 0 | 61.5 | lmo0103 |
| LMRG_00333 | hypothetical protein | 5 | 306 | 0 | 61.2 | lmo0646 |
| predicted RNA | antisense: LMRG_01626 | 3 | 183 | 0 | 61 | #N/A |
| LMRG_01633 | OsmC/Ohr family protein | 12 | 668 | 0 | 55.66666667 | lmo2199 |
| LMRG_02052 | hypothetical protein | 2 | 83 | 0 | 41.5 | lmo0953 |
| LMRG_01632 | MarR family transcriptional regulator | 69 | 2700 | 0 | 39.13043478 | lmo2200 |
| predicted RNA | antisense: LMRG_00177 | 6 | 226 | 0 | 37.66666667 | #N/A |
| LMRG_00482 | hypothetical protein | 19 | 714 | 0 | 37.57894737 | lmo0794 |
| predicted RNA | - | 14 | 476 | 0 | 34 | #N/A |
| LMRG_02677 | CtsR family transcriptional regulator | 6 | 188 | 0 | 31.33333333 | lmo0229 |
| predicted RNA | - | 8 | 244 | 0 | 30.5 | #N/A |
| LMRG_00129 | hypothetical protein | 8 | 239 | 0 | 29.875 | lmo0437 |
| LMRG_01948 | general stress protein 26 | 33 | 973 | 0 | 29.48484848 | lmo2748 |
| LMRG_00446 | hypothetical protein | 13 | 378 | 0 | 29.07692308 | lmo0758 |
| LMRG_02675 | ATP:guanido phosphotransferase | 12 | 344 | 0 | 28.66666667 | lmo0231 |
| LMRG_01674 | hypothetical protein | 59 | 1650 | 0 | 27.96610169 | lmo2158 |
| LMRG_02676 | hypothetical protein | 12 | 319 | 0 | 26.58333333 | lmo0230 |
| LMRG_00581 | Clp protease | 51 | 1311 | 0 | 25.70588235 | lmo1138 |
| LMRG_00448 | hypothetical protein | 6 | 154 | 0 | 25.66666667 | lmo0760 |
| LMRG_00137 | hypothetical protein | 15 | 374 | 0 | 24.93333333 | lmo0445 |
| LMRG_02646 | internalin C2 | 6 | 140 | 1.17E-285 | 23.33333333 | lmo0263 |
| LMRG_00447 | glyoxylase | 4 | 92 | 1.91E-252 | 23 | lmo0759 |
| predicted RNA | - | 7 | 159 | 7.63E-193 | 22.71428571 | #N/A |
| LMRG_00583 | hypothetical protein | 16 | 344 | 1.04E-243 | 21.5 | lmo1140 |
| LMRG_01626 | ATP-dependent chaperone ClpB | 80 | 1707 | 2.00E-230 | 21.3375 | lmo2206 |
| LMRG_01029 | 30S ribosomal protein S14 | 40 | 852 | 4.55E-255 | 21.3 | lmo1882 |
| LMRG_01627 | phosphoglycerate mutase | 154 | 3240 | 4.37E-233 | 21.03896104 | lmo2205 |
| LMRG_00334 | hypothetical protein | 37 | 771 | 4.93E-241 | 20.83783784 | lmo0647 |
| LMRG_02768 | hypothetical protein | 4 | 83 | 9.40E-207 | 20.75 | lmo1694 |
| LMRG_01754 | phosphate transport system regulatory protein PhoU | 5 | 102 | 6.31E-187 | 20.4 | lmo2494 |
| LMRG_00489 | hypothetical protein | 14 | 276 | 4.94E-214 | 19.71428571 | lmo0800 |
| LMRG_02611 | succinyl-diaminopimelate desuccinylase | 6 | 117 | 2.33E-182 | 19.5 | lmo0265 |
| LMRG_01764 | membrane protein | 72 | 1388 | 3.23E-219 | 19.27777778 | lmo2484 |
| LMRG_02736 | hypothetical protein | 9 | 171 | 3.09E-199 | 19 | lmo2391 |
| predicted RNA | - | 43 | 815 | 1.23E-167 | 18.95348837 | #N/A |
| predicted RNA | antisense: LMRG_00335 | 22 | 414 | 7.52E-178 | 18.81818182 | #N/A |
| LMRG_01219 | chaperonin GroS | 81 | 1484 | 4.17E-188 | 18.32098765 | lmo2069 |
| LMRG_02000 | dihydroxyacetone kinase | 4 | 72 | 1.49E-144 | 18 | lmo2697 |
| predicted RNA | antisense: LMRG_01296 | 13 | 233 | 1.77E-235 | 17.92307692 | #N/A |
| LMRG_00285 | hypothetical protein | 6 | 107 | 2.64E-116 | 17.83333333 | lmo0602 |
| LMRG_00449 | hypothetical protein | 4 | 71 | 3.23E-110 | 17.75 | lmo0761 |
| LMRG_02693 | CDF family cation efflux system protein | 22 | 389 | 1.54E-161 | 17.68181818 | lmo2575 |
| LMRG_02002 | dihydroxyacetone kinase DhaK subunit | 23 | 405 | 5.57E-165 | 17.60869565 | lmo2695 |
| predicted RNA | - | 14 | 241 | 9.22E-157 | 17.21428571 | #N/A |
| LMRG_02674 | ATP-dependent Clp protease ATP-binding subunit ClpC | 37 | 626 | 9.91E-140 | 16.91891892 | lmo0232 |
| LMRG_02808 | hypothetical protein | 7 | 118 | 2.54E-130 | 16.85714286 | lmo2132 |
| LMRG_02146 | Mg2+ transporter-C family protein | 16 | 269 | 5.53E-139 | 16.8125 | lmo2602 |
| LMRG_02218 | universal stress protein | 12 | 201 | 1.25E-136 | 16.75 | lmo2673 |
| LMRG_00666 | hypothetical protein | 14 | 234 | 4.12E-137 | 16.71428571 | lmo1220 |
| LMRG_00293 | internalin | 6 | 100 | 2.29E-133 | 16.66666667 | lmo0610 |
| LMRG_00411 | pyruvate oxidase | 42 | 697 | 3.15E-148 | 16.5952381 | lmo0722 |
| LMRG_02697 | nicotinamidase | 24 | 398 | 8.74E-139 | 16.58333333 | lmo2571 |
| LMRG_02701 | hypothetical protein | 22 | 359 | 3.17E-133 | 16.31818182 | lmo2567 |
| LMRG_01972 | DNA binding 3-demethylubiquinone-9 3-methyltransferase domain-containing protein | 51 | 784 | 3.22E-127 | 15.37254902 | lmo2724 |
| predicted RNA | antisense: LMRG_02046 | 14 | 213 | 3.84E-177 | 15.21428571 | #N/A |
| LMRG_01675 | hypothetical protein | 9 | 134 | 8.77E-110 | 14.88888889 | lmo2157 |
| LMRG_00221 | tagatose 1,6-diphosphate aldolase | 183 | 2721 | 1.34E-99 | 14.86885246 | lmo0539 |
| LMRG_01814 | glutamate decarboxylase | 33 | 484 | 1.49E-147 | 14.66666667 | lmo2434 |
| LMRG_02001 | dihydroxyacetone kinase L subunit | 16 | 233 | 1.17E-98 | 14.5625 | lmo2696 |
| predicted RNA | antisense: LMRG_02042 | 16 | 232 | 1.45E-174 | 14.5 | #N/A |
| predicted RNA | antisense: LMRG_00221 | 15 | 214 | 9.08E-169 | 14.26666667 | #N/A |
| LMRG_02003 | OsmC/Ohr family protein | 35 | 499 | 1.57E-103 | 14.25714286 | lmo0903 |
| predicted RNA | antisense: LMRG_00364 | 14 | 198 | 4.14E-134 | 14.14285714 | #N/A |
| LMRG_01780 | Clp protease | 241 | 3361 | 2.53E-101 | 13.94605809 | lmo2468 |
| predicted RNA | antisense: LMRG_02779 | 31 | 431 | 8.30E-100 | 13.90322581 | #N/A |
| LMRG_02382 | hypothetical protein | 5 | 67 | 1.75E-124 | 13.4 | lmo0133 |
| predicted RNA | - | 35 | 469 | 6.70E-92 | 13.4 | #N/A |
| predicted RNA | antisense: LMRG_00073 | 15 | 199 | 1.12E-122 | 13.26666667 | #N/A |
| predicted RNA | antisense: LMRG_00704 | 24 | 317 | 5.17E-154 | 13.20833333 | #N/A |
| LMRG_00131 | hypothetical protein | 6 | 79 | 3.36E-70 | 13.16666667 | lmo0439 |
| LMRG_01236 | peptidoglycan binding protein | 16 | 207 | 4.29E-84 | 12.9375 | lmo2085 |
| LMRG_02633 | 50S ribosomal protein L25 Ctc-form | 13 | 167 | 3.18E-71 | 12.84615385 | lmo0211 |
| predicted RNA | antisense: LMRG_01869 | 23 | 295 | 1.89E-146 | 12.82608696 | #N/A |
| predicted RNA | - | 37 | 465 | 4.87E-132 | 12.56756757 | #N/A |
| LMRG_02472 | arginine deiminase | 42 | 527 | 1.70E-94 | 12.54761905 | lmo0043 |
| LMRG_00273 | hypothetical protein | 5 | 62 | 1.74E-56 | 12.4 | lmo0591 |
| LMRG_01761 | hypothetical protein | 20 | 241 | 1.17E-69 | 12.05 | lmo2487 |
| LMRG_02147 | amidase | 10 | 120 | 3.25E-63 | 12 | lmo2603 |
| LMRG_01619 | hypothetical protein | 41 | 486 | 2.61E-69 | 11.85365854 | lmo2213 |
| LMRG_02290 | hypothetical protein | 46 | 514 | 4.62E-52 | 11.17391304 | #N/A |
| LMRG_02036 | hypothetical protein | 7 | 77 | 1.99E-69 | 11 | lmo0937 |
| LMRG_02295 | hypothetical protein | 11 | 121 | 8.24E-51 | 11 | lmo0871 |
| LMRG_01753 | phosphate ABC transporter ATP-binding protein | 13 | 142 | 2.10E-50 | 10.92307692 | lmo2495 |
| predicted RNA | - | 36 | 390 | 1.30E-88 | 10.83333333 | #N/A |
| predicted RNA | - | 23 | 247 | 1.02E-92 | 10.73913043 | #N/A |
| LMRG_01763 | PspC domain-containing protein | 46 | 485 | 9.81E-47 | 10.54347826 | lmo2485 |
| predicted RNA | antisense: LMRG_00365 | 79 | 826 | 2.26E-45 | 10.4556962 | #N/A |
| LMRG_02013 | succinate-semialdehyde dehydrogenase | 31 | 322 | 1.48E-65 | 10.38709677 | lmo0913 |
| LMRG_00341 | hypothetical protein | 81 | 831 | 1.21E-48 | 10.25925926 | lmo0654 |
| LMRG_02696 | riboflavin biosynthesis protein RibD domain-containing protein | 35 | 355 | 2.82E-47 | 10.14285714 | lmo2572 |
| LMRG_02695 | NADPH2:quinone reductase | 14 | 142 | 9.70E-42 | 10.14285714 | lmo2573 |
| LMRG_02247 | hypothetical protein | 113 | 1146 | 3.80E-61 | 10.14159292 | lmo0822 |
| LMRG_00272 | hypothetical protein | 16 | 158 | 4.99E-44 | 9.875 | lmo0590 |
| LMRG_02732 | hypothetical protein | 7 | 69 | 3.37E-37 | 9.857142857 | lmo2387 |
| LMRG_02398 | zinc transport system substrate-binding protein | 9 | 86 | 1.56E-31 | 9.555555556 | lmo0153 |
| predicted RNA | - | 30 | 285 | 1.62E-51 | 9.5 | #N/A |
| LMRG_02094 | hypothetical protein | 8 | 76 | 1.20E-33 | 9.5 | lmo0994 |
| predicted RNA | antisense: LMRG_00288 | 29 | 266 | 1.50E-55 | 9.172413793 | #N/A |
| LMRG_02622 | listeriolysin regulatory protein | 55 | 490 | 1.14E-42 | 8.909090909 | lmo0200 |
| predicted RNA | - | 128 | 1134 | 3.82E-31 | 8.859375 | #N/A |
| LMRG_02011 | hypothetical protein | 24 | 211 | 4.50E-33 | 8.791666667 | lmo0911 |
| predicted RNA | - | 36 | 315 | 5.00E-29 | 8.75 | #N/A |
| LMRG_02042 | hypothetical protein | 574 | 4865 | 2.49E-28 | 8.475609756 | lmo0944 |
| predicted RNA | - | 24 | 201 | 3.30E-25 | 8.375 | #N/A |
| LMRG_01757 | hydrolase | 58 | 475 | 2.44E-33 | 8.189655172 | lmo2491 |
| LMRG_01153 | aryl-alcohol dehydrogenase | 11 | 90 | 2.59E-25 | 8.181818182 | lmo2005 |
| LMRG_01816 | hypothetical protein | 26 | 212 | 1.29E-25 | 8.153846154 | lmo2432 |
| LMRG_01353 | hypothetical protein | 15 | 120 | 9.60E-25 | 8 | lmo1613 |
| predicted RNA | - | 26 | 207 | 6.40E-37 | 7.961538462 | #N/A |
| predicted RNA | antisense: LMRG_01149 | 29 | 229 | 1.18E-33 | 7.896551724 | #N/A |
| LMRG_02734 | hypothetical protein | 58 | 456 | 1.24E-26 | 7.862068966 | lmo2389 |
| predicted RNA | - | 36 | 277 | 1.50E-21 | 7.694444444 | #N/A |
| LMRG_00897 | transcriptional regulator ZurR | 91 | 699 | 3.06E-30 | 7.681318681 | lmo1445 |
| LMRG_02476 | hypothetical protein | 17 | 130 | 3.36E-23 | 7.647058824 | lmo0047 |
| predicted RNA | - | 82 | 622 | 1.82E-21 | 7.585365854 | #N/A |
| LMRG_02738 | hypothetical protein | 14 | 106 | 9.18E-22 | 7.571428571 | lmo2393 |
| LMRG_01680 | thioredoxin | 99 | 742 | 6.82E-26 | 7.494949495 | lmo2152 |
| predicted RNA | - | 104 | 778 | 4.76E-23 | 7.480769231 | #N/A |
| LMRG_01444 | hypothetical protein | 111 | 823 | 2.31E-27 | 7.414414414 | lmo1526 |
| LMRG_01755 | HTH-type transcriptional repressor czrA | 25 | 185 | 2.83E-19 | 7.4 | lmo2493 |
| LMRG_02579 | hypothetical protein | 284 | 2051 | 5.97E-09 | 7.221830986 | lmo0292 |
| predicted RNA | antisense: LMRG_00364 | 33 | 235 | 8.76E-27 | 7.121212121 | #N/A |
| LMRG_00357 | hypothetical protein | 10 | 71 | 8.56E-18 | 7.1 | lmo0669 |
| LMRG_02415 | hypothetical protein | 174 | 1233 | 2.19E-17 | 7.086206897 | lmo0170 |
| predicted RNA | antisense: LMRG_02779 | 31 | 216 | 4.02E-31 | 6.967741935 | #N/A |
| LMRG_01296 | zinc transport system substrate-binding protein | 339 | 2339 | 7.35E-14 | 6.899705015 | lmo1671 |
| LMRG_00928 | heat-inducible transcription repressor HrcA | 139 | 954 | 1.44E-19 | 6.863309353 | lmo1475 |
| LMRG_00292 | hypothetical protein | 38 | 259 | 2.51E-19 | 6.815789474 | lmo0609 |
| LMRG_01737 | sigma-54 modulation protein | 92 | 619 | 9.23E-23 | 6.72826087 | lmo2511 |
| LMRG_02884 | secreted protein | 18 | 121 | 4.42E-17 | 6.722222222 | lmo0479 |
| LMRG_00926 | chaperone DnaK | 276 | 1849 | 6.39E-07 | 6.699275362 | lmo1473 |
| LMRG_01028 | hypothetical protein | 81 | 537 | 1.75E-17 | 6.62962963 | lmo1881 |
| LMRG_02679 | hypothetical protein | 10 | 66 | 3.04E-17 | 6.6 | lmo2588 |
| predicted RNA | - | 31 | 202 | 2.61E-26 | 6.516129032 | #N/A |
| LMRG_02215 | hypothetical protein | 14 | 91 | 4.97E-16 | 6.5 | lmo2670 |
| LMRG_02239 | hypothetical protein | 269 | 1736 | 1.84E-15 | 6.453531599 | lmo2692 |
| LMRG_02731 | hypothetical protein | 13 | 83 | 5.22E-14 | 6.384615385 | lmo2386 |
| LMRG_01140 | alpha-acetolactate decarboxylase | 347 | 2173 | 1.58E-12 | 6.262247839 | lmo1992 |
| LMRG_00521 | hypothetical protein | 17 | 106 | 2.08E-13 | 6.235294118 | lmo1059 |
| predicted RNA | - | 133 | 829 | 5.00E-14 | 6.233082707 | #N/A |
| LMRG_00743 | glycerol-3-phosphate dehydrogenase | 32 | 198 | 5.21E-18 | 6.1875 | lmo1293 |
| LMRG_01431 | glycerol uptake facilitator protein | 12 | 73 | 6.40E-12 | 6.083333333 | lmo1539 |
| predicted RNA | - | 56 | 340 | 7.37E-17 | 6.071428571 | #N/A |
| LMRG_01762 | hypothetical protein | 31 | 188 | 2.63E-16 | 6.064516129 | lmo2486 |
| LMRG_01218 | chaperonin GroL | 281 | 1684 | 5.23E-06 | 5.992882562 | lmo2068 |
| LMRG_02694 | hypothetical protein | 241 | 1407 | 4.30E-14 | 5.838174274 | lmo2574 |
| LMRG_01756 | hypothetical protein | 338 | 1964 | 2.88E-13 | 5.810650888 | lmo2492 |
| predicted RNA | - | 168 | 971 | 2.98E-13 | 5.779761905 | #N/A |
| LMRG_02700 | hypothetical protein | 14 | 80 | 2.05E-11 | 5.714285714 | lmo2568 |
| LMRG_01573 | hypothetical protein | 14 | 79 | 1.95E-09 | 5.642857143 | lmo2258 |
| LMRG_02331 | hypothetical protein | 24 | 133 | 3.16E-10 | 5.541666667 | lmo0083 |
| LMRG_00472 | mannose-specific PTS system IIA component | 21 | 116 | 1.38E-10 | 5.523809524 | lmo0784 |
| LMRG_01815 | tributyrin esterase | 120 | 658 | 1.48E-10 | 5.483333333 | lmo2433 |
| LMRG_00679 | thioredoxin | 104 | 569 | 2.18E-12 | 5.471153846 | lmo1233 |
| LMRG_02248 | aldo/keto reductase | 111 | 604 | 2.23E-10 | 5.441441441 | lmo0823 |
| LMRG_02813 | hypothetical protein | 13 | 70 | 3.75E-09 | 5.384615385 | lmo1789 |
| predicted RNA | antisense: LMRG_00288 | 144 | 768 | 7.93E-11 | 5.333333333 | #N/A |
| LMRG_02609 | hypothetical protein | 25 | 131 | 1.77E-10 | 5.24 | lmo0267 |
| LMRG_02055 | N-acetylglucosamine-6-phosphate deacetylase | 168 | 880 | 5.95E-09 | 5.238095238 | lmo0956 |
| LMRG_01656 | hypothetical protein | 17 | 89 | 1.72E-09 | 5.235294118 | lmo2176 |
| LMRG_01794 | hypothetical protein | 13 | 68 | 9.42E-10 | 5.230769231 | lmo2454 |
| LMRG_02812 | MarR family transcriptional regulator | 39 | 202 | 6.79E-10 | 5.179487179 | lmo1788 |
| LMRG_01384 | thiol peroxidase | 43 | 222 | 4.11E-10 | 5.162790698 | lmo1583 |
| LMRG_00345 | endonuclease III | 13 | 66 | 1.24E-08 | 5.076923077 | lmo0658 |
| LMRG_00879 | osmoprotectant transport system permease | 43 | 218 | 2.60E-10 | 5.069767442 | lmo1427 |
| LMRG_02056 | glucosamine-6-phosphate isomerase | 59 | 299 | 8.90E-12 | 5.06779661 | lmo0957 |
| predicted RNA | antisense: LMRG_02397 | 80 | 403 | 8.77E-09 | 5.0375 | #N/A |
| LMRG_05029 | Met tRNA | 81 | 403 | 5.84E-09 | 4.975308642 | #N/A |
| LMRG_00878 | osmoprotectant transport system substrate-binding protein | 79 | 391 | 4.62E-09 | 4.949367089 | lmo1426 |
| LMRG_01641 | arsenate reductase | 24 | 118 | 1.05E-07 | 4.916666667 | lmo2191 |
| LMRG_02054 | hypothetical protein | 50 | 243 | 8.38E-11 | 4.86 | lmo0955 |
| LMRG_01491 | HTH-type transcriptional regulator ytlI | 27 | 131 | 1.45E-08 | 4.851851852 | lmo2352 |
| LMRG_01795 | hypothetical protein | 161 | 779 | 2.32E-08 | 4.838509317 | lmo2453 |
| predicted RNA | antisense: LMRG_02397 | 53 | 256 | 1.65E-08 | 4.830188679 | #N/A |
| LMRG_01822 | arsenate reductase | 72 | 346 | 9.16E-09 | 4.805555556 | lmo2426 |
| predicted RNA | antisense: LMRG_01678 | 93 | 446 | 3.44E-08 | 4.795698925 | #N/A |
| LMRG_00469 | mannose-specific PTS system IID component | 13 | 62 | 1.15E-08 | 4.769230769 | lmo0781 |
| LMRG_02397 | peptide/nickel transport system substrate-binding protein | 1002 | 4775 | 0.005247719 | 4.765469062 | lmo0152 |
| LMRG_00488 | hypothetical protein | 107 | 509 | 3.27E-08 | 4.757009346 | lmo0799 |
| LMRG_00826 | peptidase T-like protein | 62 | 294 | 2.14E-08 | 4.741935484 | lmo1375 |
| LMRG_00877 | osmoprotectant transport system permease | 43 | 203 | 7.10E-09 | 4.720930233 | lmo1425 |
| predicted RNA | antisense: LMRG_01840 LMRG_01839 | 272 | 1280 | 5.55E-09 | 4.705882353 | #N/A |
| LMRG_01066 | hypothetical protein | 73 | 340 | 3.46E-10 | 4.657534247 | lmo1919 |
| LMRG_01679 | ribonucleotide reductase-associated flavodoxin | 204 | 949 | 1.84E-07 | 4.651960784 | lmo2153 |
| LMRG_00470 | mannose-specific PTS system IIC component | 43 | 200 | 7.17E-09 | 4.651162791 | lmo0782 |
| LMRG_00294 | FMN-dependent NADH-azoreductase 1 | 24 | 111 | 2.83E-07 | 4.625 | lmo0611 |
| LMRG_01469 | hypothetical protein | 95 | 436 | 2.53E-08 | 4.589473684 | lmo1501 |
| LMRG_00132 | hypothetical protein | 50 | 227 | 1.15E-07 | 4.54 | lmo0440 |
| LMRG_00925 | chaperone DnaJ | 105 | 471 | 3.15E-07 | 4.485714286 | lmo1472 |
| LMRG_02764 | hypothetical protein | 228 | 1021 | 2.47E-06 | 4.478070175 | lmo1690 |
| LMRG_01207 | protoheme IX farnesyltransferase | 15 | 67 | 1.93E-06 | 4.466666667 | lmo2057 |
| LMRG_01068 | hypothetical protein | 24 | 107 | 3.84E-07 | 4.458333333 | lmo1921 |
| predicted RNA | - | 47 | 209 | 2.45E-11 | 4.446808511 | #N/A |
| LMRG_00885 | glutathione reductase | 18 | 80 | 1.03E-06 | 4.444444444 | lmo1433 |
| LMRG_02076 | hypothetical protein | 18 | 80 | 3.98E-06 | 4.444444444 | lmo0976 |
| LMRG_00290 | ABC transporter | 70 | 311 | 4.35E-07 | 4.442857143 | lmo0607 |
| predicted RNA | antisense: LMRG_01556 | 60 | 266 | 2.64E-06 | 4.433333333 | #N/A |
| LMRG_01154 | acetolactate synthase catabolic | 85 | 376 | 2.83E-07 | 4.423529412 | lmo2006 |
| LMRG_00927 | co-chaperone GrpE | 74 | 326 | 9.01E-09 | 4.405405405 | lmo1474 |
| LMRG_02350 | hypothetical protein | 164 | 704 | 9.58E-09 | 4.292682927 | lmo0101 |
| LMRG_02320 | sigma-B negative regulator | 24 | 103 | 2.85E-06 | 4.291666667 | lmo0896 |
| LMRG_00836 | hypothetical protein | 207 | 885 | 8.45E-06 | 4.275362319 | lmo1384 |
| LMRG_00880 | osmoprotectant transport system ATP-binding protein | 62 | 264 | 2.14E-06 | 4.258064516 | lmo1428 |
| predicted RNA | antisense: LMRG_02384 | 72 | 306 | 6.76E-06 | 4.25 | #N/A |
| predicted RNA | - | 42 | 177 | 2.49E-09 | 4.214285714 | #N/A |
| LMRG_02053 | hypothetical protein | 32 | 133 | 8.57E-06 | 4.15625 | lmo0954 |
| LMRG_02666 | ribonuclease III family protein | 25 | 103 | 1.17E-05 | 4.12 | lmo0240 |
| predicted RNA | antisense: LMRG_02397 | 54 | 221 | 7.03E-06 | 4.092592593 | #N/A |
| LMRG_00891 | superoxide dismutase | 110 | 450 | 2.97E-06 | 4.090909091 | lmo1439 |
| LMRG_00289 | transcription regulator MarR family protein | 183 | 748 | 8.08E-06 | 4.087431694 | lmo0606 |
| LMRG_02216 | hypothetical protein | 16 | 65 | 2.31E-05 | 4.0625 | lmo2671 |
| LMRG_01690 | hypothetical protein | 34 | 138 | 2.03E-06 | 4.058823529 | lmo2557 |
| LMRG_01850 | phosphatidylglycerophosphatase A | 76 | 307 | 9.10E-07 | 4.039473684 | lmo2398 |
| LMRG_02758 | hypothetical protein | 26 | 105 | 9.71E-06 | 4.038461538 | lmo1684 |
| LMRG_02845 | GntR family transcriptional regulator | 34 | 137 | 6.75E-06 | 4.029411765 | lmo0902 |
| LMRG_00291 | ABC transporter | 152 | 610 | 1.44E-04 | 4.013157895 | lmo0608 |
| LMRG_02414 | sugar uptake protein | 28 | 112 | 1.18E-05 | 4 | lmo0169 |
| predicted RNA | antisense: LMRG_05509 | 42 | 167 | 3.33E-06 | 3.976190476 | #N/A |
| LMRG_01770 | thioredoxin-disulfide reductase | 90 | 356 | 2.26E-05 | 3.955555556 | lmo2478 |
| LMRG_02869 | mannose-specific PTS system IIB component | 17 | 67 | 8.85E-05 | 3.941176471 | lmo0783 |
| predicted RNA | antisense: LMRG_05515 | 42 | 165 | 5.69E-06 | 3.928571429 | #N/A |
| LMRG_00261 | hypothetical protein | 56 | 220 | 1.95E-05 | 3.928571429 | lmo0579 |
| LMRG_01851 | thioredoxin-like protein | 23 | 89 | 1.88E-05 | 3.869565217 | lmo2397 |
| LMRG_01678 | ribonucleoside-diphosphate reductase | 617 | 2371 | 0.039824699 | 3.842787682 | lmo2154 |
| LMRG_02667 | cysteinyl-tRNA synthetase | 54 | 207 | 3.60E-05 | 3.833333333 | lmo0239 |
| LMRG_02665 | TrmH family RNA methyltransferase group 3 | 63 | 241 | 1.51E-06 | 3.825396825 | lmo0241 |
| LMRG_00884 | hypothetical protein | 337 | 1276 | 2.19E-04 | 3.786350148 | lmo1432 |
| LMRG_02082 | deblocking aminopeptidase | 22 | 83 | 5.76E-05 | 3.772727273 | lmo0982 |
| LMRG_00266 | hypothetical protein | 135 | 508 | 5.74E-05 | 3.762962963 | lmo0584 |
| LMRG_00707 | hypothetical protein | 54 | 203 | 2.40E-06 | 3.759259259 | lmo1258 |
| predicted RNA | antisense: LMRG_02771 | 49 | 184 | 7.32E-08 | 3.755102041 | #N/A |
| LMRG_00032 | hypothetical protein | 27 | 101 | 1.99E-04 | 3.740740741 | lmo0341 |
| LMRG_05008 | Arg tRNA | 50 | 187 | 1.45E-04 | 3.74 | #N/A |
| LMRG_00898 | zinc transport system permease | 61 | 228 | 1.63E-05 | 3.737704918 | lmo1446 |
| LMRG_02663 | RNA polymerase sporulation-specific sigma factor | 181 | 673 | 1.27E-04 | 3.718232044 | lmo0243 |
| LMRG_01937 | hypothetical protein | 21 | 78 | 9.21E-05 | 3.714285714 | lmo2759 |
| LMRG_00278 | hypothetical protein | 78 | 289 | 7.49E-06 | 3.705128205 | lmo0596 |
| LMRG_02786 | carbonic anhydrase | 33 | 122 | 8.27E-05 | 3.696969697 | lmo0811 |
| LMRG_01114 | hypothetical protein | 153 | 558 | 3.50E-04 | 3.647058824 | lmo1967 |
| LMRG_01297 | hypothetical protein | 117 | 425 | 5.59E-05 | 3.632478632 | lmo1670 |
| predicted RNA | antisense: LMRG_00515 LMRG_00514 | 119 | 430 | 1.75E-04 | 3.613445378 | #N/A |
| LMRG_01870 | hypothetical protein | 53 | 190 | 2.12E-04 | 3.58490566 | lmo2828 |
| LMRG_00337 | membrane protein | 31 | 111 | 2.04E-04 | 3.580645161 | lmo0650 |
| LMRG_02349 | hypothetical protein | 169 | 605 | 6.05E-05 | 3.579881657 | lmo0100 |
| LMRG_00753 | cell division suppressor protein yneA | 19 | 68 | 5.66E-04 | 3.578947368 | lmo1303 |
| LMRG_00745 | host factor-I protein | 243 | 867 | 5.76E-05 | 3.567901235 | lmo1295 |
| LMRG_02959 | multidrug resistance protein | 37 | 131 | 1.00E-05 | 3.540540541 | lmo1712 |
| predicted RNA | antisense: LMRG_01609 | 75 | 262 | 5.43E-04 | 3.493333333 | #N/A |
| LMRG_00835 | isopentenyl-diphosphate delta-isomerase type 2 | 292 | 1019 | 0.002406516 | 3.489726027 | lmo1383 |
| LMRG_02063 | hypothetical protein | 70 | 244 | 1.20E-04 | 3.485714286 | lmo0964 |
| LMRG_02317 | anti-sigma-B factor antagonist | 77 | 267 | 1.85E-04 | 3.467532468 | lmo0893 |
| LMRG_00354 | antibiotic transport system ATP-binding protein | 82 | 282 | 4.34E-04 | 3.43902439 | lmo0667 |
| LMRG_00899 | zinc uptake system ATP-binding protein zurA | 55 | 189 | 3.94E-05 | 3.436363636 | lmo1447 |
| LMRG_01823 | glycine cleavage system H protein | 37 | 127 | 4.03E-04 | 3.432432432 | lmo2425 |
| LMRG_02050 | hypothetical protein | 38 | 130 | 2.17E-04 | 3.421052632 | lmo0951 |
| LMRG_01811 | hypothetical protein | 56 | 191 | 4.20E-04 | 3.410714286 | lmo2437 |
| LMRG_00258 | hypothetical protein | 18 | 61 | 3.96E-04 | 3.388888889 | lmo0576 |
| LMRG_02643 | type III pantothenate kinase | 149 | 504 | 5.73E-04 | 3.382550336 | lmo0221 |
| LMRG_02644 | chaperonin HslO | 41 | 138 | 1.68E-04 | 3.365853659 | lmo0222 |
| LMRG_01556 | gp27 | 114 | 383 | 4.07E-04 | 3.359649123 | #N/A |
| LMRG_02714 | general stress protein 13 | 143 | 480 | 6.94E-05 | 3.356643357 | lmo2369 |
| LMRG_01615 | hypothetical protein | 87 | 292 | 3.01E-04 | 3.356321839 | lmo2217 |
| LMRG_02735 | hypothetical protein | 159 | 527 | 7.10E-04 | 3.314465409 | lmo2390 |
| LMRG_02445 | cytochrome aa3 quinol oxidase subunit IV | 31 | 102 | 0.00194844 | 3.290322581 | lmo0016 |
| LMRG_00520 | hypothetical protein | 58 | 190 | 0.001066446 | 3.275862069 | lmo1058 |
| LMRG_01365 | hypothetical protein | 81 | 265 | 2.92E-04 | 3.271604938 | lmo1602 |
| predicted RNA | antisense: LMRG_02224 | 73 | 238 | 0.001730138 | 3.260273973 | #N/A |
| LMRG_01796 | carboxylesterase | 47 | 153 | 4.39E-04 | 3.255319149 | lmo2452 |
| predicted RNA | antisense: LMRG_05511 | 75 | 244 | 0.001100033 | 3.253333333 | #N/A |
| LMRG_01357 | thioredoxin-like protein ytpP | 29 | 93 | 0.003804507 | 3.206896552 | lmo1609 |
| LMRG_02718 | cellobiose-specific PTS system IIB component | 31 | 99 | 0.002545682 | 3.193548387 | lmo2373 |
| LMRG_00235 | hypothetical protein | 410 | 1307 | 0.006174283 | 3.187804878 | lmo0553 |
| LMRG_02293 | hypothetical protein | 22 | 70 | 0.001841892 | 3.181818182 | lmo0869 |
| predicted RNA | antisense: LMRG_02834 | 111 | 353 | 0.001722101 | 3.18018018 | #N/A |
| LMRG_02245 | hypothetical protein | 29 | 92 | 0.001924661 | 3.172413793 | lmo0820 |
| LMRG_05032 | Arg tRNA | 58 | 184 | 0.002078209 | 3.172413793 | #N/A |
| predicted RNA | antisense: LMRG_01571 | 112 | 355 | 0.001936706 | 3.169642857 | #N/A |
| LMRG_00103 | pyruvate,water dikinase | 25 | 79 | 0.001765837 | 3.16 | lmo0410 |
| LMRG_02041 | starvation-inducible DNA-binding protein | 846 | 2651 | 0.029780284 | 3.13356974 | lmo0943 |
| LMRG_01113 | hypothetical protein | 94 | 293 | 0.001053115 | 3.117021277 | lmo1966 |
| LMRG_02007 | hypothetical protein | 83 | 258 | 0.001066446 | 3.108433735 | lmo0907 |
| LMRG_02057 | GntR family transcriptional regulator | 48 | 149 | 0.001066276 | 3.104166667 | lmo0958 |
| LMRG_02610 | hypothetical protein | 31 | 96 | 0.002731204 | 3.096774194 | lmo0266 |
| LMRG_01575 | protease I | 32 | 99 | 0.002100988 | 3.09375 | lmo2256 |
| LMRG_02263 | MarR family transcriptional regulator | 32 | 99 | 0.002865296 | 3.09375 | lmo0840 |
| LMRG_01833 | FeS assembly ATPase SufC | 278 | 857 | 0.006208014 | 3.082733813 | lmo2415 |
| LMRG_02437 | diamine N-acetyltransferase | 33 | 101 | 0.002572121 | 3.060606061 | lmo0009 |
| LMRG_02792 | hypothetical protein | 76 | 231 | 0.001558446 | 3.039473684 | #N/A |
| predicted RNA | - | 73 | 220 | 0.00451781 | 3.01369863 | #N/A |
| LMRG_02375 | hypothetical protein | 33 | 99 | 0.004438201 | 3 | lmo0126 |
| LMRG_00417 | hypothetical protein | 45 | 133 | 0.004725323 | 2.955555556 | lmo0729 |
| LMRG_01568 | hypothetical protein | 42 | 124 | 0.002673939 | 2.952380952 | lmo2263 |
| predicted RNA | - | 88 | 256 | 0.005176832 | 2.909090909 | #N/A |
| LMRG_02343 | hypothetical protein | 21 | 61 | 0.008216044 | 2.904761905 | lmo0094 |
| predicted RNA | - | 82 | 236 | 0.003455037 | 2.87804878 | #N/A |
| LMRG_02077 | hypothetical protein | 37 | 106 | 0.00581421 | 2.864864865 | lmo0977 |
| LMRG_02938 | serine O-acetyltransferase | 22 | 63 | 0.013315824 | 2.863636364 | lmo0238 |
| predicted RNA | antisense: LMRG_00233 | 117 | 333 | 0.008711523 | 2.846153846 | #N/A |
| LMRG_01576 | hypothetical protein | 37 | 105 | 0.017136024 | 2.837837838 | lmo2255 |
| LMRG_01402 | DNA polymerase I | 55 | 156 | 0.005065534 | 2.836363636 | lmo1565 |
| LMRG_02117 | PTS system glucose-specific transporter subunit IIA | 82 | 230 | 0.0032698 | 2.804878049 | lmo1017 |
| LMRG_00080 | hypothetical protein | 209 | 584 | 0.010165448 | 2.794258373 | lmo0387 |
| LMRG_02274 | hypothetical protein | 99 | 275 | 0.010550408 | 2.777777778 | lmo0851 |
| LMRG_01777 | NADPH dehydrogenase | 31 | 86 | 0.006728628 | 2.774193548 | lmo2471 |
| predicted RNA | antisense: LMRG_05504 | 63 | 174 | 0.018568862 | 2.761904762 | #N/A |
| LMRG_02089 | hypothetical protein | 57 | 157 | 0.013197896 | 2.754385965 | lmo0989 |
| LMRG_00336 | hypothetical protein | 23 | 63 | 0.025737754 | 2.739130435 | lmo0649 |
| predicted RNA | antisense: LMRG_05508 | 87 | 237 | 0.012951781 | 2.724137931 | #N/A |
| LMRG_00236 | hypothetical protein | 89 | 242 | 0.018535735 | 2.719101124 | lmo0554 |
| LMRG_02259 | hypothetical protein | 109 | 295 | 0.003559122 | 2.706422018 | lmo0836 |
| LMRG_00512 | polypeptide deformylase | 243 | 649 | 0.008885988 | 2.670781893 | lmo1051 |
| LMRG_00081 | hypothetical protein | 48 | 128 | 0.018674629 | 2.666666667 | lmo0388 |
| LMRG_00173 | transcription regulator LysR | 29 | 77 | 0.018961544 | 2.655172414 | lmo0492 |
| LMRG_01007 | peptide-methionine (S)-S-oxide reductase | 482 | 1278 | 0.041761156 | 2.651452282 | lmo1860 |
| predicted RNA | antisense: LMRG_05517 | 97 | 257 | 0.014391515 | 2.649484536 | #N/A |
| predicted RNA | antisense: LMRG_00479 | 94 | 248 | 0.023529652 | 2.638297872 | #N/A |
| LMRG_02673 | DNA repair protein RadA | 41 | 108 | 0.010760569 | 2.634146341 | lmo0233 |
| LMRG_01624 | hypothetical protein | 63 | 165 | 0.005014105 | 2.619047619 | lmo2208 |
| LMRG_00519 | L-lactate dehydrogenase | 79 | 206 | 0.021438437 | 2.607594937 | lmo1057 |
| LMRG_00458 | transcriptional regulator | 25 | 65 | 0.018961544 | 2.6 | lmo0770 |
| LMRG_01623 | hypothetical protein | 25 | 65 | 0.021265447 | 2.6 | lmo2209 |
| LMRG_01403 | formamidopyrimidine-DNA glycosylase | 34 | 88 | 0.021453834 | 2.588235294 | lmo1564 |
| predicted RNA | antisense: LMRG_05505 | 123 | 317 | 0.025246604 | 2.577235772 | #N/A |
| LMRG_00701 | hypothetical protein | 178 | 457 | 0.02523123 | 2.56741573 | #N/A |
| LMRG_02615 | HlyD family secretion protein | 39 | 100 | 0.021634833 | 2.564102564 | lmo0193 |
| predicted RNA | antisense: LMRG_05514 | 96 | 245 | 0.03036087 | 2.552083333 | #N/A |
| LMRG_01055 | hypothetical protein | 42 | 107 | 0.043428349 | 2.547619048 | lmo1908 |
| predicted RNA | antisense: LMRG_00554 | 67 | 170 | 8.34E-04 | 2.537313433 | #N/A |
| LMRG_02244 | hypothetical protein | 89 | 225 | 0.030493736 | 2.528089888 | lmo0819 |
| LMRG_01069 | hypothetical protein | 42 | 106 | 0.01320132 | 2.523809524 | lmo1922 |
| LMRG_00205 | hypothetical protein | 48 | 121 | 0.033431119 | 2.520833333 | lmo0524 |
| LMRG_00090 | hypothetical protein | 52 | 131 | 0.032359701 | 2.519230769 | lmo0397 |
| LMRG_00442 | hypothetical protein | 54 | 136 | 0.019786837 | 2.518518519 | lmo0754 |
| LMRG_02037 | protein-tyrosine phosphatase | 157 | 392 | 0.033431119 | 2.496815287 | lmo0938 |
| predicted RNA | - | 238 | 591 | 0.026337976 | 2.483193277 | #N/A |
| LMRG_02386 | peptide/nickel transport system permease | 146 | 362 | 0.020624817 | 2.479452055 | lmo0137 |
| LMRG_02635 | peptidyl-tRNA hydrolase | 54 | 131 | 0.039822953 | 2.425925926 | lmo0213 |
| LMRG_00280 | transcription regulator CRP/FNR family protein | 26 | 63 | 0.044916888 | 2.423076923 | lmo0597 |
| LMRG_01847 | hydrolase | 43 | 104 | 0.032798066 | 2.418604651 | lmo2401 |
| LMRG_01856 | transcriptional regulator | 46 | 111 | 0.016880131 | 2.413043478 | lmo2842 |
| LMRG_01036 | hypothetical protein | 107 | 255 | 0.033198142 | 2.38317757 | lmo1889 |
| LMRG_01051 | hypothetical protein | 47 | 112 | 0.020005519 | 2.382978723 | lmo1904 |
| LMRG_02049 | hypothetical protein | 134 | 319 | 0.044879257 | 2.380597015 | lmo0950 |
| LMRG_02946 | hypothetical protein | 252 | 595 | 0.021633399 | 2.361111111 | #N/A |
| LMRG_02108 | hypothetical protein | 252 | 586 | 0.041761156 | 2.325396825 | lmo1008 |
| LMRG_00452 | lipoate-protein ligase A | 37 | 86 | 0.038929387 | 2.324324324 | lmo0764 |
| LMRG_01834 | FeS assembly protein SufD | 118 | 273 | 0.042967549 | 2.313559322 | lmo2414 |
| LMRG_01047 | aspartate 1-decarboxylase | 341 | 784 | 0.044283805 | 2.299120235 | lmo1900 |
| LMRG_01054 | dihydrodipicolinate reductase | 64 | 147 | 0.02980491 | 2.296875 | lmo1907 |
| LMRG_02444 | cytochrome aa3 quinol oxidase subunit III | 64 | 145 | 0.038929387 | 2.265625 | lmo0015 |
| predicted RNA | - | 353 | 799 | 0.04803035 | 2.263456091 | #N/A |
| LMRG_01210 | hypothetical protein | 76 | 169 | 0.035520736 | 2.223684211 | lmo2060 |
| LMRG_02727 | multicomponent Na+:H+ antiporter subunit E | 124 | 63 | 0.032125345 | 0.508064516 | lmo2382 |
| LMRG_02008 | hypothetical protein | 65 | 33 | 0.020005519 | 0.507692308 | lmo0908 |
| LMRG_01700 | homoserine dehydrogenase | 201 | 101 | 0.047175323 | 0.502487562 | lmo2547 |
| LMRG_02800 | sodium transport system permease | 48 | 24 | 0.035658352 | 0.5 | lmo2140 |
| LMRG_02585 | aminotransferase | 52 | 26 | 0.038929387 | 0.5 | lmo0286 |
| LMRG_02250 | hydroxymethylglutaryl-CoA reductase | 48 | 24 | 0.03981199 | 0.5 | lmo0825 |
| LMRG_00681 | aspartate kinase | 182 | 91 | 0.040562131 | 0.5 | lmo1235 |
| LMRG_02620 | UDP-N-acetylglucosamine diphosphorylase/glucosamine-1-phosphate N-acetyltransferase | 130 | 65 | 0.04364084 | 0.5 | lmo0198 |
| LMRG_01394 | acetyl-CoA carboxylase carboxyl transferase subunit beta | 48 | 24 | 0.044283805 | 0.5 | lmo1573 |
| LMRG_02124 | hypothetical protein | 44 | 22 | 0.047789583 | 0.5 | lmo1024 |
| LMRG_02312 | hypothetical protein | 283 | 141 | 0.04803035 | 0.498233216 | lmo0888 |
| LMRG_00784 | secreted protein | 257 | 128 | 0.04215241 | 0.498054475 | lmo1334 |
| LMRG_00413 | hypothetical protein | 350 | 173 | 0.039495623 | 0.494285714 | lmo0724 |
| LMRG_01002 | D-alanyl-D-alanine carboxypeptidase | 81 | 40 | 0.023124458 | 0.49382716 | lmo1855 |
| predicted RNA | - | 1606 | 790 | 0.033748261 | 0.491905355 | #N/A |
| LMRG_02641 | tRNA(Ile)-lysidine synthetase | 147 | 72 | 0.03871668 | 0.489795918 | lmo0219 |
| LMRG_01347 | D-amino acid aminotransferase | 333 | 163 | 0.03990536 | 0.489489489 | lmo1619 |
| LMRG_00908 | hypothetical protein | 190 | 93 | 0.002408231 | 0.489473684 | lmo1456 |
| LMRG_00995 | manganese transport system membrane protein mntC | 178 | 87 | 0.029152211 | 0.488764045 | lmo1848 |
| LMRG_00537 | teichoic acids export ATP-binding protein tagH | 84 | 41 | 0.020653577 | 0.488095238 | lmo1075 |
| LMRG_02621 | ribose-phosphate pyrophosphokinase 1 | 355 | 173 | 0.038289935 | 0.487323944 | lmo0199 |
| LMRG_01143 | deoxyribose-phosphate aldolase | 142 | 69 | 0.037493838 | 0.485915493 | lmo1995 |
| LMRG_00883 | hypothetical protein | 266 | 129 | 0.041730146 | 0.484962406 | lmo1431 |
| LMRG_02502 | phosphoribosylformylglycinamidine synthase II | 31 | 15 | 0.021382699 | 0.483870968 | lmo1769 |
| LMRG_02167 | 30S ribosomal protein S17 | 461 | 223 | 0.034746604 | 0.48373102 | lmo2623 |
| LMRG_01773 | phosphoglucomutase/phosphomannomutase | 54 | 26 | 0.03885516 | 0.481481481 | lmo2475 |
| LMRG_01892 | hypothetical protein | 52 | 25 | 0.016954793 | 0.480769231 | lmo2804 |
| LMRG_00993 | multidrug resistance protein norM | 50 | 24 | 0.015638073 | 0.48 | lmo1846 |
| LMRG_00685 | rdgB/HAM1 family non-canonical purine NTP pyrophosphatase | 221 | 106 | 0.026237749 | 0.479638009 | lmo1239 |
| predicted RNA | - | 1672 | 800 | 0.013365301 | 0.4784689 | #N/A |
| LMRG_01697 | hypothetical protein | 46 | 22 | 0.034356578 | 0.47826087 | lmo2550 |
| LMRG_02180 | 1,4-dihydroxy-2-naphthoate octaprenyltransferase | 67 | 32 | 0.018674629 | 0.47761194 | lmo2635 |
| LMRG_02684 | response regulator | 74 | 35 | 0.023677106 | 0.472972973 | lmo2583 |
| LMRG_02879 | hypothetical protein | 55 | 26 | 0.009199536 | 0.472727273 | lmo0528 |
| LMRG_01168 | isoleucyl-tRNA synthetase | 70 | 33 | 5.73E-04 | 0.471428571 | lmo2019 |
| LMRG_00536 | teichoic acid transport system permease | 51 | 24 | 0.029891732 | 0.470588235 | lmo1074 |
| LMRG_00965 | ribulose-phosphate 3-epimerase | 149 | 70 | 0.026728806 | 0.469798658 | lmo1818 |
| LMRG_02252 | pyruvate:ferredoxin oxidoreductase | 66 | 31 | 0.026347347 | 0.46969697 | lmo0829 |
| LMRG_01406 | replication initiation and membrane attachment protein | 330 | 155 | 0.035630534 | 0.46969697 | lmo1561 |
| LMRG_01374 | NifS/icsS protein | 49 | 23 | 0.013354675 | 0.469387755 | lmo1593 |
| predicted RNA | - | 868 | 406 | 0.020408525 | 0.467741935 | #N/A |
| LMRG_00211 | N-acetylglucosaminyltransferase | 30 | 14 | 0.044813253 | 0.466666667 | lmo0529 |
| LMRG_00281 | biotin biosynthesis protein BioY | 133 | 62 | 0.010993339 | 0.466165414 | #N/A |
| LMRG_01793 | phosphopyruvate hydratase | 1243 | 579 | 0.02779667 | 0.465808528 | lmo2455 |
| LMRG_00301 | hypothetical protein | 41 | 19 | 0.021453834 | 0.463414634 | lmo0618 |
| LMRG_01330 | ABC transporter ATP-binding protein | 237 | 109 | 0.021382699 | 0.459915612 | lmo1636 |
| LMRG_01284 | hypothetical protein | 192 | 88 | 0.023052624 | 0.458333333 | lmo2130 |
| LMRG_01373 | septation ring formation regulator EzrA | 313 | 143 | 0.030958788 | 0.45686901 | lmo1594 |
| LMRG_00905 | hypothetical protein | 125 | 57 | 0.007034615 | 0.456 | lmo1453 |
| LMRG_01063 | beta-lactamase | 145 | 66 | 0.015455111 | 0.455172414 | lmo1916 |
| LMRG_01685 | hypothetical protein | 33 | 15 | 0.047803696 | 0.454545455 | lmo2147 |
| LMRG_00758 | hypothetical protein | 97 | 44 | 0.008216044 | 0.453608247 | lmo1308 |
| LMRG_00703 | alpha-phosphotrehalase | 181 | 82 | 0.02165941 | 0.453038674 | lmo1254 |
| LMRG_02699 | peptide/nickel transport system substrate-binding protein | 371 | 168 | 0.032359701 | 0.452830189 | lmo2569 |
| LMRG_02168 | 50S ribosomal protein L29 | 349 | 158 | 0.009456532 | 0.452722063 | lmo2624 |
| LMRG_02150 | DNA-directed RNA polymerase subunit alpha | 685 | 310 | 0.045972061 | 0.452554745 | lmo2606 |
| LMRG_00952 | ribonuclease III | 250 | 113 | 0.011835797 | 0.452 | lmo1805 |
| LMRG_01062 | malate dehydrogenase | 113 | 51 | 0.014960466 | 0.451327434 | lmo1915 |
| LMRG_02185 | heptaprenyl diphosphate synthase component I | 246 | 111 | 0.018334167 | 0.451219512 | lmo2640 |
| LMRG_00534 | pyruvate carboxylase | 404 | 182 | 0.038260918 | 0.45049505 | lmo1072 |
| LMRG_00961 | hypothetical protein | 60 | 27 | 0.014081458 | 0.45 | lmo1814 |
| LMRG_02820 | hypothetical protein | 145 | 65 | 0.0383802 | 0.448275862 | lmo1796 |
| predicted RNA | antisense: LMRG_02657 | 654 | 293 | 0.03871668 | 0.448012232 | #N/A |
| LMRG_01034 | N6-adenine-specific DNA methylase | 76 | 34 | 0.005810536 | 0.447368421 | lmo1887 |
| LMRG_02656 | large subunit ribosomal protein L10 | 1275 | 569 | 0.032743346 | 0.44627451 | lmo0250 |
| LMRG_01253 | glutamine amidotransferase subunit pdxT | 299 | 133 | 0.009044345 | 0.444816054 | lmo2102 |
| LMRG_00941 | nicotinate nucleotide adenylyltransferase | 135 | 60 | 0.006294116 | 0.444444444 | lmo1488 |
| LMRG_00765 | di-trans,poly-cis-decaprenylcistransferase | 187 | 83 | 0.013134209 | 0.443850267 | lmo1315 |
| LMRG_02051 | hypothetical protein | 210 | 93 | 0.006922704 | 0.442857143 | lmo0952 |
| LMRG_02360 | hypothetical protein | 113 | 50 | 0.004666342 | 0.442477876 | lmo0111 |
| LMRG_02181 | thiamine biosynthesis lipoprotein | 118 | 52 | 0.016805534 | 0.440677966 | lmo2636 |
| LMRG_02599 | hypothetical protein | 66 | 29 | 0.007640077 | 0.439393939 | lmo0276 |
| LMRG_02177 | 30S ribosomal protein S10 | 746 | 327 | 0.013197896 | 0.438337802 | lmo2633 |
| LMRG_02712 | glucose-6-phosphate isomerase | 963 | 422 | 0.037643237 | 0.438213915 | lmo2367 |
| LMRG_02088 | peptide chain release factor 3 | 176 | 77 | 0.013046027 | 0.4375 | lmo0988 |
| LMRG_01408 | threonyl-tRNA synthetase | 112 | 49 | 0.015305858 | 0.4375 | lmo1559 |
| LMRG_02432 | hypothetical protein | 117 | 51 | 0.031589071 | 0.435897436 | lmo0004 |
| LMRG_00802 | hypothetical protein | 590 | 254 | 0.006832118 | 0.430508475 | lmo1352 |
| LMRG_00550 | hypothetical protein | 135 | 58 | 0.007061656 | 0.42962963 | lmo1088 |
| LMRG_00147 | hypothetical protein | 35 | 15 | 0.004337716 | 0.428571429 | lmo0455 |
| LMRG_02348 | hypothetical protein | 138 | 59 | 0.004955323 | 0.427536232 | lmo0099 |
| LMRG_00771 | hypothetical protein | 134 | 57 | 0.004312974 | 0.425373134 | lmo1321 |
| LMRG_02278 | D-alanine--D-alanine ligase | 146 | 62 | 0.005807016 | 0.424657534 | lmo0855 |
| LMRG_01638 | peptide/nickel transport system permease | 721 | 305 | 0.04919203 | 0.423023578 | lmo2194 |
| LMRG_02501 | phosphoribosylformylglycinamidine synthase I | 31 | 13 | 0.049232295 | 0.419354839 | lmo1770 |
| LMRG_05006 | Gly tRNA | 5433 | 2278 | 0.010459305 | 0.419289527 | #N/A |
| LMRG_01772 | aldose 1-epimerase | 456 | 191 | 0.010762338 | 0.418859649 | lmo2476 |
| LMRG_00744 | tRNA delta(2)-isopentenylpyrophosphate transferase | 203 | 85 | 0.004477661 | 0.418719212 | lmo1294 |
| LMRG_00944 | ribosome biogenesis GTPase YqeH | 696 | 291 | 0.049164568 | 0.418103448 | lmo1491 |
| LMRG_01303 | methionine adenosyltransferase | 137 | 57 | 0.004283812 | 0.416058394 | lmo1664 |
| LMRG_01473 | uridine kinase | 41 | 17 | 0.04301591 | 0.414634146 | lmo1497 |
| LMRG_01360 | DNA segregation ATPase FtsK/SpoIIIE | 152 | 63 | 0.010095766 | 0.414473684 | lmo1606 |
| LMRG_00704 | trehalose-specific PTS system IIBC component | 162 | 67 | 0.0063181 | 0.413580247 | lmo1255 |
| LMRG_01038 | recombination protein U | 179 | 74 | 0.005263869 | 0.413407821 | lmo1891 |
| LMRG_00275 | formate/nitrite transporter | 119 | 49 | 0.001306163 | 0.411764706 | lmo0593 |
| LMRG_02176 | 50S ribosomal protein L3 | 506 | 207 | 0.006832118 | 0.409090909 | lmo2632 |
| LMRG_00870 | nucleotide pyrophosphatase | 93 | 38 | 0.008061528 | 0.408602151 | lmo1418 |
| LMRG_00966 | ribosome small subunit-dependent GTPase A | 142 | 58 | 0.008512739 | 0.408450704 | lmo1819 |
| LMRG_00901 | endonuclease 4 | 130 | 53 | 0.006954465 | 0.407692308 | lmo1449 |
| LMRG_01124 | ribonuclease Z | 32 | 13 | 0.030716609 | 0.40625 | lmo1977 |
| predicted RNA | antisense: LMRG_01720 | 160 | 65 | 0.04102053 | 0.40625 | #N/A |
| LMRG_00674 | ribonuclease HIII | 32 | 13 | 0.043051938 | 0.40625 | lmo1228 |
| LMRG_02280 | alpha/beta fold family hydrolase | 106 | 43 | 0.001883725 | 0.405660377 | lmo0857 |
| LMRG_05034 | Gly tRNA | 4718 | 1889 | 0.005916195 | 0.400381518 | #N/A |
| LMRG_01084 | ribosome-associated GTPase EngA | 200 | 80 | 0.004192147 | 0.4 | lmo1937 |
| LMRG_00954 | 3-oxoacyl-ACP reductase | 45 | 18 | 0.014038157 | 0.4 | lmo1807 |
| LMRG_01714 | F-type H+-transporting ATPase subunit C | 484 | 193 | 0.001306163 | 0.398760331 | lmo2534 |
| LMRG_00529 | GTP-binding protein TypA/BipA | 224 | 89 | 0.006238853 | 0.397321429 | lmo1067 |
| LMRG_01169 | cell division initiation protein | 68 | 27 | 0.014408909 | 0.397058824 | lmo2020 |
| LMRG_01582 | PiT family inorganic phosphate transporter | 754 | 298 | 0.017102841 | 0.395225464 | lmo2249 |
| LMRG_00773 | hypothetical protein | 947 | 373 | 0.003406903 | 0.393875396 | lmo1323 |
| LMRG_02271 | polar amino acid transport system ATP-binding protein | 112 | 44 | 0.001070677 | 0.392857143 | lmo0848 |
| LMRG_00832 | hypothetical protein | 396 | 155 | 0.001640079 | 0.391414141 | lmo1380 |
| LMRG_00318 | 2-haloalkanoic acid dehalogenase | 296 | 114 | 0.002014978 | 0.385135135 | lmo0635 |
| LMRG_02770 | hypothetical protein | 65 | 25 | 0.007034615 | 0.384615385 | lmo1696 |
| LMRG_01926 | antibiotic transport system ATP-binding protein | 39 | 15 | 0.012232232 | 0.384615385 | lmo2769 |
| LMRG_01340 | hypothetical protein | 136 | 52 | 0.001194099 | 0.382352941 | lmo1626 |
| LMRG_05045 | Lys tRNA | 1810 | 690 | 0.00345001 | 0.38121547 | #N/A |
| LMRG_01099 | diaminopimelate decarboxylase | 108 | 41 | 0.002777291 | 0.37962963 | lmo1952 |
| LMRG_01079 | trans-hexaprenyltranstransferase | 467 | 177 | 0.002933682 | 0.379014989 | lmo1932 |
| LMRG_01090 | hypothetical protein | 45 | 17 | 0.005014008 | 0.377777778 | lmo1943 |
| LMRG_02586 | D-methionine transport system substrate-binding protein | 398 | 150 | 0.002200451 | 0.376884422 | lmo0285 |
| LMRG_02315 | serine/threonine-protein kinase rsbT | 146 | 55 | 7.89E-04 | 0.376712329 | lmo0891 |
| LMRG_02119 | hypothetical protein | 77 | 29 | 6.28E-04 | 0.376623377 | lmo1019 |
| LMRG_00657 | integral membrane protein | 40 | 15 | 0.036269745 | 0.375 | lmo1211 |
| LMRG_02175 | 50S ribosomal protein L4/L1 | 884 | 331 | 0.002853719 | 0.374434389 | lmo2631 |
| LMRG_01243 | glycine betaine transporter | 59 | 22 | 3.96E-04 | 0.372881356 | lmo2092 |
| LMRG_00419 | hypothetical protein | 43 | 16 | 0.039856044 | 0.372093023 | lmo0731 |
| LMRG_01363 | peroxiredoxin | 250 | 93 | 0.002708912 | 0.372 | lmo1604 |
| LMRG_01792 | 2,3-bisphosphoglycerate-independent phosphoglycerate mutase | 1390 | 517 | 0.002493673 | 0.371942446 | lmo2456 |
| LMRG_00812 | exodeoxyribonuclease VII small subunit | 119 | 44 | 0.012379762 | 0.369747899 | lmo1362 |
| LMRG_00612 | threonine-phosphate decarboxylase | 38 | 14 | 0.00563131 | 0.368421053 | lmo1169 |
| LMRG_05036 | Lys tRNA | 3699 | 1354 | 0.001375696 | 0.366044877 | #N/A |
| LMRG_01414 | delta-aminolevulinic acid dehydratase | 41 | 15 | 0.004441472 | 0.365853659 | lmo1554 |
| predicted RNA | antisense: LMRG_01081 | 707 | 257 | 0.006238853 | 0.363507779 | #N/A |
| LMRG_00392 | hypothetical protein | 295 | 107 | 0.0016435 | 0.362711864 | lmo0703 |
| LMRG_02759 | glutamate-1-semialdehyde 2,1-aminomutase 2 | 91 | 33 | 5.05E-04 | 0.362637363 | lmo1685 |
| LMRG_01412 | porphobilinogen deaminase | 50 | 18 | 0.002877714 | 0.36 | lmo1556 |
| predicted RNA | - | 225 | 81 | 0.011560716 | 0.36 | #N/A |
| LMRG_02744 | multiple sugar transport system permease | 53 | 19 | 0.002233874 | 0.358490566 | lmo0179 |
| LMRG_02784 | fructokinase | 90 | 32 | 1.69E-04 | 0.355555556 | lmo0813 |
| LMRG_01443 | bifunctional preprotein translocase subunit SecD/SecF | 249 | 88 | 0.001306163 | 0.353413655 | lmo1527 |
| predicted RNA | - | 2015 | 710 | 3.40E-04 | 0.35235732 | #N/A |
| predicted RNA | - | 375 | 132 | 4.83E-05 | 0.352 | #N/A |
| LMRG_01141 | pyrimidine-nucleoside phosphorylase | 37 | 13 | 0.002721735 | 0.351351351 | lmo1993 |
| LMRG_01033 | carboxypeptidase Taq | 180 | 63 | 8.59E-04 | 0.35 | lmo1886 |
| LMRG_01717 | ATP synthase F1 alpha subunit | 277 | 96 | 9.11E-04 | 0.346570397 | lmo2531 |
| LMRG_00171 | shikimate 5-dehydrogenase | 52 | 18 | 0.002151164 | 0.346153846 | lmo0490 |
| LMRG_01691 | fructose-16-bisphosphate aldolase class II | 2798 | 968 | 0.001156859 | 0.345961401 | lmo2556 |
| LMRG_01630 | 3-oxoacyl-ACP synthase | 67 | 23 | 2.92E-04 | 0.343283582 | lmo2202 |
| LMRG_01696 | transcription termination factor Rho | 35 | 12 | 0.001999326 | 0.342857143 | lmo2551 |
| LMRG_01097 | segregation and condensation protein B | 794 | 272 | 7.23E-04 | 0.34256927 | lmo1950 |
| LMRG_01425 | septum site-determining protein minC | 38 | 13 | 0.006680086 | 0.342105263 | lmo1545 |
| LMRG_00852 | acetyltransferase | 328 | 112 | 3.96E-04 | 0.341463415 | lmo1400 |
| LMRG_01385 | site-specific DNA-methyltransferase | 484 | 163 | 5.43E-04 | 0.33677686 | lmo1582 |
| LMRG_02473 | 30S ribosomal protein S6 | 520 | 175 | 4.20E-04 | 0.336538462 | lmo0044 |
| predicted RNA | - | 4219 | 1411 | 0.003535268 | 0.334439441 | #N/A |
| predicted RNA | - | 560 | 187 | 0.001804117 | 0.333928571 | #N/A |
| LMRG_02187 | serine/threonine protein phosphatase | 105 | 35 | 3.64E-04 | 0.333333333 | lmo2642 |
| LMRG_01230 | hypothetical protein | 42 | 14 | 9.19E-04 | 0.333333333 | lmo2079 |
| LMRG_01382 | protease IV | 36 | 12 | 0.002942662 | 0.333333333 | lmo1585 |
| LMRG_01195 | 2-dehydropantoate 2-reductase | 30 | 10 | 0.004772802 | 0.333333333 | lmo2046 |
| LMRG_01790 | phosphoglycerate kinase | 686 | 228 | 5.42E-04 | 0.332361516 | lmo2458 |
| LMRG_01098 | ScpA/B protein | 103 | 34 | 2.76E-05 | 0.330097087 | lmo1951 |
| LMRG_01059 | hypothetical protein | 73 | 24 | 3.33E-05 | 0.328767123 | lmo1912 |
| predicted RNA | - | 229 | 75 | 0.039007579 | 0.327510917 | #N/A |
| LMRG_00736 | DNA topoisomerase IV B subunit | 513 | 168 | 0.005301755 | 0.32748538 | lmo1286 |
| LMRG_01058 | hypothetical protein | 43 | 14 | 8.40E-04 | 0.325581395 | lmo1911 |
| LMRG_01997 | aldo/keto reductase family oxidoreductase | 83 | 27 | 4.52E-05 | 0.325301205 | lmo2700 |
| LMRG_02122 | response regulator LiaR | 163 | 53 | 1.52E-05 | 0.325153374 | lmo1022 |
| LMRG_00084 | hypothetical protein | 114 | 37 | 0.00194844 | 0.324561404 | lmo0391 |
| LMRG_00762 | hypothetical protein | 37 | 12 | 0.027913956 | 0.324324324 | lmo1312 |
| LMRG_00085 | hypothetical protein | 130 | 42 | 5.72E-06 | 0.323076923 | lmo0392 |
| LMRG_01441 | hypothetical protein | 78 | 25 | 0.003860523 | 0.320512821 | lmo1529 |
| LMRG_02900 | beta-glucoside operon transcriptional antiterminator | 94 | 30 | 8.10E-06 | 0.319148936 | lmo2773 |
| LMRG_01713 | ATP synthase F0 A subunit | 421 | 134 | 1.23E-04 | 0.318289786 | lmo2535 |
| LMRG_02658 | 50S ribosomal protein L11 | 2141 | 680 | 5.50E-04 | 0.317608594 | lmo0248 |
| LMRG_00287 | hypothetical protein | 41 | 13 | 0.019268586 | 0.317073171 | lmo0604 |
| LMRG_00871 | hypothetical protein | 121 | 38 | 5.23E-06 | 0.314049587 | lmo1419 |
| LMRG_00475 | amino acid transporter AAT family protein | 287 | 90 | 9.08E-05 | 0.31358885 | lmo0787 |
| predicted RNA | - | 354 | 111 | 0.003199764 | 0.313559322 | #N/A |
| LMRG_02425 | membrane protein OxaA | 428 | 134 | 6.66E-05 | 0.313084112 | lmo2854 |
| LMRG_00759 | hypothetical protein | 48 | 15 | 0.004644073 | 0.3125 | lmo1309 |
| LMRG_02516 | glutamyl-tRNA(Gln) amidotransferase subunit A | 308 | 96 | 1.24E-04 | 0.311688312 | lmo1755 |
| LMRG_00163 | cfr family radical SAM enzyme | 289 | 90 | 7.62E-05 | 0.311418685 | lmo0482 |
| LMRG_00749 | glutamine synthetase type I | 621 | 191 | 1.08E-04 | 0.307568438 | lmo1299 |
| LMRG_02485 | adenylosuccinate synthetase | 154 | 47 | 2.18E-05 | 0.305194805 | lmo0055 |
| LMRG_00942 | hypothetical protein | 59 | 18 | 0.009270389 | 0.305084746 | lmo1489 |
| LMRG_00216 | hypothetical protein | 69 | 21 | 5.69E-06 | 0.304347826 | lmo0534 |
| LMRG_00774 | hypothetical protein | 793 | 241 | 8.03E-06 | 0.303909206 | lmo1324 |
| LMRG_02868 | AAT family amino acid transporter | 33 | 10 | 4.88E-04 | 0.303030303 | lmo0798 |
| LMRG_00377 | hypothetical protein | 33 | 10 | 0.001095668 | 0.303030303 | lmo0689 |
| LMRG_01981 | ABC transporter CydDC cysteine exporter CydC | 308 | 93 | 5.50E-05 | 0.301948052 | lmo2715 |
| LMRG_02279 | UDP-N-acetylmuramoylalanyl-D-glutamyl-2 | 170 | 51 | 7.76E-06 | 0.3 | lmo0856 |
| LMRG_01891 | hypothetical protein | 47 | 14 | 0.004544726 | 0.29787234 | lmo2805 |
| LMRG_01637 | peptide/nickel transport system permease | 1132 | 336 | 7.25E-04 | 0.296819788 | lmo2195 |
| LMRG_02751 | hypothetical protein | 118 | 35 | 7.56E-06 | 0.296610169 | lmo0186 |
| LMRG_00955 | malonyl CoA-acyl carrier protein transacylase | 98 | 29 | 2.02E-06 | 0.295918367 | lmo1808 |
| LMRG_00223 | iron complex transport system substrate-binding protein | 122 | 36 | 1.29E-06 | 0.295081967 | lmo0541 |
| LMRG_00288 | hypothetical protein | 75 | 22 | 3.80E-06 | 0.293333333 | lmo0605 |
| LMRG_01979 | cytochrome d ubiquinol oxidase subunit II | 580 | 170 | 2.49E-05 | 0.293103448 | lmo2717 |
| LMRG_02153 | 50S ribosomal protein L36 | 3824 | 1111 | 1.42E-05 | 0.290533473 | lmo2609 |
| LMRG_01252 | pyridoxine biosynthesis protein | 341 | 99 | 1.11E-05 | 0.290322581 | lmo2101 |
| LMRG_01182 | cell division protein FtsA | 428 | 124 | 1.93E-05 | 0.289719626 | lmo2033 |
| LMRG_02671 | 2-C-methyl-D-erythritol 4-phosphate cytidylyltransferase | 2304 | 665 | 2.72E-04 | 0.288628472 | lmo0235 |
| predicted RNA | antisense: LMRG_01430 LMRG_01429 | 427 | 123 | 0.001841892 | 0.288056206 | #N/A |
| LMRG_02114 | glycine betaine/proline transport system ATP-binding protein | 293 | 84 | 9.67E-06 | 0.28668942 | lmo1014 |
| LMRG_00263 | SAM-dependent methyltransferase | 155 | 44 | 1.07E-05 | 0.283870968 | lmo0581 |
| LMRG_05004 | Lys tRNA | 2329 | 660 | 7.03E-06 | 0.283383426 | #N/A |
| LMRG_01489 | hypothetical protein | 120 | 34 | 1.09E-05 | 0.283333333 | lmo2354 |
| LMRG_01771 | UDP-glucose 4-epimerase | 110 | 31 | 3.42E-07 | 0.281818182 | lmo2477 |
| LMRG_02510 | neurotransmitter:Na+ symporter | 64 | 18 | 3.12E-07 | 0.28125 | lmo1761 |
| LMRG_01242 | argininosuccinate lyase | 25 | 7 | 3.35E-04 | 0.28 | lmo2091 |
| LMRG_01331 | DNA binding 3-demethylubiquinone-9 3-methyltransferase domain-containing protein | 193 | 54 | 3.19E-07 | 0.279792746 | lmo1635 |
| LMRG_02081 | hypothetical protein | 175 | 48 | 6.10E-07 | 0.274285714 | lmo0981 |
| LMRG_01866 | glycerate kinase | 77 | 21 | 1.60E-07 | 0.272727273 | lmo2832 |
| predicted RNA | - | 837 | 228 | 2.67E-05 | 0.272401434 | #N/A |
| LMRG_01718 | ATP synthase F1 gamma subunit | 788 | 213 | 2.31E-06 | 0.270304569 | lmo2530 |
| LMRG_01018 | phosphomannomutase | 37 | 10 | 1.37E-05 | 0.27027027 | lmo1871 |
| LMRG_02183 | NADH dehydrogenase | 592 | 160 | 5.06E-05 | 0.27027027 | lmo2638 |
| LMRG_01013 | phosphotransferase | 26 | 7 | 0.00230137 | 0.269230769 | lmo1866 |
| LMRG_02798 | hypothetical protein | 272 | 73 | 2.06E-06 | 0.268382353 | lmo2142 |
| predicted RNA | - | 358 | 96 | 0.011052388 | 0.268156425 | #N/A |
| LMRG_02864 | hypothetical protein | 64 | 17 | 0.001034586 | 0.265625 | #N/A |
| LMRG_00810 | hypothetical protein | 57 | 15 | 2.40E-05 | 0.263157895 | lmo1360 |
| LMRG_02626 | actin-assembly inducing protein ActA | 153 | 40 | 1.42E-07 | 0.261437908 | lmo0204 |
| LMRG_01364 | aminopeptidase | 153 | 40 | 2.03E-07 | 0.261437908 | lmo1603 |
| LMRG_01003 | purine nucleoside phosphorylase | 81 | 21 | 2.60E-06 | 0.259259259 | lmo1856 |
| LMRG_00381 | flagellar motor switch protein FliN/FliY | 722 | 185 | 2.34E-07 | 0.256232687 | lmo0693 |
| LMRG_02710 | CTP synthase | 594 | 152 | 6.70E-07 | 0.255892256 | lmo2559 |
| LMRG_01341 | hypothetical protein | 43 | 11 | 7.57E-07 | 0.255813953 | lmo1625 |
| LMRG_05050 | His tRNA | 232 | 59 | 6.68E-04 | 0.254310345 | #N/A |
| predicted RNA | - | 284 | 72 | 0.005807016 | 0.253521127 | #N/A |
| predicted RNA | - | 222 | 56 | 0.005060871 | 0.252252252 | #N/A |
| LMRG_01488 | hypothetical protein | 282 | 71 | 1.21E-07 | 0.25177305 | lmo2355 |
| LMRG_00553 | hypothetical protein | 64 | 16 | 7.57E-07 | 0.25 | lmo1091 |
| LMRG_00393 | hypothetical protein | 128 | 32 | 2.24E-06 | 0.25 | lmo0704 |
| LMRG_02496 | AIR carboxylase | 40 | 10 | 3.98E-04 | 0.25 | lmo1775 |
| LMRG_02070 | D-alanine transfer protein | 1034 | 255 | 2.17E-05 | 0.246615087 | lmo0971 |
| LMRG_02872 | hypothetical protein | 1602 | 393 | 8.01E-08 | 0.245318352 | lmo0694 |
| predicted RNA | - | 213 | 52 | 0.001184674 | 0.244131455 | #N/A |
| LMRG_02827 | translation initiation factor IF-3 | 4106 | 997 | 2.11E-06 | 0.242815392 | lmo1785 |
| predicted RNA | - | 5311 | 1286 | 3.68E-08 | 0.242138957 | #N/A |
| LMRG_00384 | hypothetical protein | 2429 | 588 | 3.52E-07 | 0.242074928 | lmo0695 |
| LMRG_00843 | simple sugar transport system permease | 87 | 21 | 4.89E-09 | 0.24137931 | lmo1391 |
| LMRG_01659 | hypothetical protein | 29 | 7 | 1.10E-05 | 0.24137931 | lmo2173 |
| LMRG_00772 | transcription termination factor NusA | 287 | 69 | 2.34E-09 | 0.240418118 | lmo1322 |
| predicted RNA | - | 887 | 213 | 1.16E-07 | 0.240135287 | #N/A |
| LMRG_01712 | ATP synthase I | 150 | 36 | 2.41E-07 | 0.24 | lmo2536 |
| LMRG_00394 | flagellar hook-associated protein FlgK | 573 | 137 | 3.41E-08 | 0.239092496 | lmo0705 |
| predicted RNA | antisense: LMRG_01792 | 176 | 42 | 8.59E-04 | 0.238636364 | #N/A |
| LMRG_02307 | membrane protein | 202 | 48 | 1.26E-09 | 0.237623762 | lmo0883 |
| LMRG_00819 | phosphate butyryltransferase | 131 | 31 | 7.29E-11 | 0.236641221 | lmo1369 |
| LMRG_00811 | exodeoxyribonuclease VII large subunit | 89 | 21 | 4.10E-10 | 0.235955056 | lmo1361 |
| LMRG_01867 | beta-phosphoglucomutase | 51 | 12 | 1.08E-05 | 0.235294118 | lmo2831 |
| predicted RNA | - | 290 | 68 | 0.001100033 | 0.234482759 | #N/A |
| LMRG_02321 | SulP family sulfate permease | 64 | 15 | 1.99E-10 | 0.234375 | lmo0897 |
| LMRG_02978 | hypothetical protein | 64 | 15 | 0.001125382 | 0.234375 | #N/A |
| LMRG_01636 | peptide/nickel transport system substrate-binding protein | 2391 | 560 | 1.75E-07 | 0.234211627 | lmo2196 |
| LMRG_00987 | pyrimidine operon attenuation protein/uracil phosphoribosyltransferase | 47 | 11 | 6.92E-05 | 0.234042553 | lmo1840 |
| LMRG_01978 | cytochrome bd-I oxidase subunit I | 159 | 37 | 1.15E-08 | 0.232704403 | lmo2718 |
| LMRG_00803 | hypothetical protein | 86 | 20 | 5.80E-09 | 0.23255814 | lmo1353 |
| LMRG_02136 | CRISPR-associated protein cas2 | 56 | 13 | 3.17E-04 | 0.232142857 | #N/A |
| predicted RNA | - | 358 | 83 | 0.001804117 | 0.231843575 | #N/A |
| LMRG_01583 | hypothetical protein | 986 | 228 | 8.19E-09 | 0.231237323 | lmo2248 |
| LMRG_01213 | hypothetical protein | 39 | 9 | 7.00E-04 | 0.230769231 | lmo2063 |
| LMRG_01676 | hypothetical protein | 39 | 9 | 7.11E-04 | 0.230769231 | lmo2156 |
| LMRG_02071 | D-alanine--poly(phosphoribitol) ligase subunit 2 | 1124 | 259 | 7.20E-10 | 0.230427046 | lmo0972 |
| LMRG_02426 | ribonuclease P | 211 | 48 | 4.46E-09 | 0.227488152 | lmo2855 |
| LMRG_01741 | cell division ATP-binding protein FtsE | 721 | 163 | 6.66E-10 | 0.226074896 | lmo2507 |
| LMRG_00981 | dihydroorotate dehydrogenase electron transfer subunit | 49 | 11 | 3.31E-06 | 0.224489796 | lmo1834 |
| LMRG_01743 | D-glutamyl-L-m-Dpm peptidase P45 | 1944 | 436 | 9.47E-08 | 0.224279835 | lmo2505 |
| LMRG_00956 | fatty acid/phospholipid synthesis protein PlsX | 90 | 20 | 1.29E-10 | 0.222222222 | lmo1809 |
| predicted RNA | - | 389 | 86 | 3.72E-05 | 0.221079692 | #N/A |
| predicted RNA | - | 242 | 53 | 2.11E-04 | 0.219008264 | #N/A |
| predicted RNA | - | 256 | 56 | 2.67E-04 | 0.21875 | #N/A |
| LMRG_01788 | transcriptional regulator | 719 | 156 | 3.22E-10 | 0.216968011 | lmo2460 |
| LMRG_00957 | fatty acid biosynthesis transcriptional regulator | 598 | 129 | 4.58E-12 | 0.215719064 | lmo1810 |
| LMRG_01577 | AGZA family MFS transporter xanthine/uracil permease | 182 | 39 | 2.34E-10 | 0.214285714 | lmo2254 |
| LMRG_01453 | nitrogen regulatory protein P-II | 33 | 7 | 5.43E-04 | 0.212121212 | lmo1517 |
| LMRG_00391 | hypothetical protein | 496 | 105 | 3.72E-11 | 0.211693548 | lmo0702 |
| LMRG_02060 | protease | 177 | 37 | 7.11E-12 | 0.209039548 | lmo0961 |
| LMRG_02270 | glutamine ABC transporter | 144 | 30 | 2.32E-13 | 0.208333333 | lmo0847 |
| predicted RNA | - | 221 | 46 | 7.94E-06 | 0.208144796 | #N/A |
| LMRG_00087 | extracellular P60 protein | 135 | 28 | 2.80E-11 | 0.207407407 | lmo0394 |
| LMRG_00151 | hypothetical protein | 92 | 19 | 6.63E-08 | 0.206521739 | #N/A |
| LMRG_01742 | cell division transport system permease | 762 | 157 | 2.04E-11 | 0.206036745 | lmo2506 |
| LMRG_00535 | iron complex transport system substrate-binding protein | 68 | 14 | 2.93E-09 | 0.205882353 | lmo1073 |
| predicted RNA | - | 787 | 162 | 4.55E-09 | 0.205844981 | #N/A |
| predicted RNA | - | 254 | 52 | 7.39E-06 | 0.204724409 | #N/A |
| LMRG_00386 | flagellar hook protein FlgE | 362 | 74 | 8.09E-12 | 0.20441989 | lmo0697 |
| LMRG_02607 | oligopeptide transport system permease oppC | 54 | 11 | 2.17E-12 | 0.203703704 | lmo0269 |
| LMRG_00370 | flagellar basal-body rod protein FlgG | 54 | 11 | 2.43E-07 | 0.203703704 | lmo0682 |
| LMRG_01980 | ABC transporter CydDC cysteine exporter CydD | 430 | 87 | 1.19E-11 | 0.202325581 | lmo2716 |
| LMRG_01073 | chorismate mutase | 195 | 39 | 4.57E-11 | 0.2 | lmo1926 |
| LMRG_01428 | 50S ribosomal protein L21 | 6089 | 1215 | 3.38E-08 | 0.199540154 | lmo1542 |
| LMRG_00395 | flagellar hook-associated protein 3 | 517 | 101 | 2.40E-13 | 0.195357834 | lmo0706 |
| LMRG_02773 | hypothetical protein | 385 | 75 | 4.01E-15 | 0.194805195 | lmo1699 |
| LMRG_02134 | hypothetical protein | 423 | 82 | 1.41E-16 | 0.193853428 | lmo2594 |
| predicted RNA | - | 301 | 58 | 9.22E-06 | 0.19269103 | #N/A |
| LMRG_02073 | D-alanine--poly(phosphoribitol) ligase subunit 1 | 270 | 52 | 2.81E-14 | 0.192592593 | lmo0974 |
| predicted RNA | antisense: LMRG_01691 | 203 | 39 | 1.42E-05 | 0.192118227 | #N/A |
| LMRG_01472 | O-methyltransferase | 73 | 14 | 1.24E-09 | 0.191780822 | lmo1498 |
| predicted RNA | - | 335 | 64 | 1.11E-05 | 0.191044776 | #N/A |
| LMRG_02624 | listeriolysin O | 168 | 32 | 1.15E-13 | 0.19047619 | lmo0202 |
| LMRG_00398 | hypothetical protein | 2607 | 494 | 7.01E-14 | 0.189489835 | lmo0709 |
| predicted RNA | antisense: LMRG_02182 | 431 | 81 | 9.73E-15 | 0.187935035 | #N/A |
| LMRG_02783 | enoyl-(acyl carrier protein) reductase | 256 | 48 | 5.67E-15 | 0.1875 | lmo0814 |
| LMRG_01791 | triosephosphate isomerase | 659 | 123 | 9.75E-15 | 0.186646434 | lmo2457 |
| LMRG_00761 | hypothetical protein | 70 | 13 | 2.10E-12 | 0.185714286 | lmo1311 |
| LMRG_00389 | flagellar motor switch protein FliN/FliY | 890 | 165 | 2.56E-14 | 0.185393258 | lmo0700 |
| LMRG_01744 | hypothetical protein | 65 | 12 | 5.56E-12 | 0.184615385 | lmo2504 |
| predicted RNA | - | 378 | 68 | 2.00E-06 | 0.17989418 | #N/A |
| LMRG_00388 | flagellar motor switch protein FliM | 1078 | 191 | 2.38E-16 | 0.177179963 | lmo0699 |
| LMRG_00953 | acyl carrier protein | 362 | 64 | 1.58E-14 | 0.17679558 | lmo1806 |
| predicted RNA | - | 665 | 117 | 1.11E-10 | 0.17593985 | #N/A |
| LMRG_00104 | hypothetical protein | 74 | 13 | 2.12E-11 | 0.175675676 | lmo0412 |
| LMRG_00215 | ACT domain-containing protein | 74 | 13 | 2.26E-07 | 0.175675676 | lmo0533 |
| LMRG_00390 | hypothetical protein | 274 | 48 | 5.67E-19 | 0.175182482 | lmo0701 |
| LMRG_00662 | bax protein | 190 | 33 | 7.49E-21 | 0.173684211 | lmo1216 |
| LMRG_01508 | hypothetical protein | 1747 | 301 | 1.15E-14 | 0.172295363 | lmo2335 |
| LMRG_00404 | flagellar assembly protein H | 2207 | 379 | 3.22E-17 | 0.171726325 | lmo0715 |
| LMRG_02138 | CRISPR-associated protein | 47 | 8 | 1.46E-21 | 0.170212766 | #N/A |
| predicted RNA | - | 259 | 44 | 7.88E-07 | 0.16988417 | #N/A |
| predicted RNA | - | 282 | 47 | 2.32E-07 | 0.166666667 | #N/A |
| LMRG_00365 | flagellar biosynthetic protein FliQ | 571 | 95 | 4.06E-24 | 0.166374781 | lmo0677 |
| LMRG_00407 | hypothetical protein | 319 | 53 | 5.84E-24 | 0.166144201 | lmo0718 |
| LMRG_02746 | multiple sugar transport system substrate-binding protein | 103 | 17 | 3.42E-21 | 0.165048544 | lmo0181 |
| LMRG_00385 | flagellar hook capping protein | 1003 | 165 | 2.51E-22 | 0.164506481 | lmo0696 |
| LMRG_01715 | ATP synthase F0 subunit B | 1436 | 236 | 6.64E-20 | 0.164345404 | lmo2533 |
| LMRG_02238 | autolysin | 1956 | 321 | 1.78E-16 | 0.164110429 | lmo2691 |
| predicted RNA | - | 476 | 78 | 1.71E-10 | 0.163865546 | #N/A |
| LMRG_00476 | hypothetical protein | 55 | 9 | 3.69E-21 | 0.163636364 | lmo0788 |
| LMRG_02379 | hypothetical protein | 110 | 18 | 9.32E-20 | 0.163636364 | lmo0130 |
| predicted RNA | - | 385 | 63 | 6.88E-08 | 0.163636364 | #N/A |
| LMRG_01487 | hypothetical protein | 44 | 7 | 2.13E-06 | 0.159090909 | #N/A |
| predicted RNA | - | 1512 | 236 | 6.50E-18 | 0.156084656 | #N/A |
| LMRG_02059 | hypothetical protein | 154 | 24 | 2.94E-25 | 0.155844156 | lmo0960 |
| predicted RNA | - | 2547 | 383 | 7.86E-27 | 0.150372988 | #N/A |
| LMRG_00840 | ABC transporter | 293 | 44 | 1.28E-24 | 0.150170648 | lmo1388 |
| predicted RNA | - | 181 | 27 | 1.95E-09 | 0.149171271 | #N/A |
| predicted RNA | - | 222 | 33 | 1.25E-09 | 0.148648649 | #N/A |
| LMRG_02072 | membrane protein | 622 | 92 | 6.60E-26 | 0.147909968 | lmo0973 |
| predicted RNA | - | 325 | 48 | 1.43E-10 | 0.147692308 | #N/A |
| LMRG_02623 | 1-phosphatidylinositol phosphodiesterase | 299 | 44 | 3.76E-31 | 0.147157191 | lmo0201 |
| LMRG_02306 | hypothetical protein | 367 | 54 | 2.22E-31 | 0.147138965 | lmo0882 |
| LMRG_00859 | pyruvate formate-lyase activating enzyme | 48 | 7 | 1.99E-11 | 0.145833333 | lmo1407 |
| LMRG_02137 | CRISPR-associated protein cas1 | 69 | 10 | 4.98E-17 | 0.144927536 | #N/A |
| LMRG_00045 | long-chain fatty acid CoA ligase (AMP-binding) | 152 | 22 | 2.06E-33 | 0.144736842 | lmo0354 |
| LMRG_00402 | flagellar M-ring protein FliF | 1309 | 189 | 4.43E-19 | 0.144385027 | lmo0713 |
| LMRG_01507 | 1-phosphofructokinase | 791 | 111 | 3.77E-29 | 0.140328698 | lmo2336 |
| LMRG_01506 | hypothetical protein | 679 | 95 | 9.72E-33 | 0.139911635 | lmo2337 |
| LMRG_05005 | Leu tRNA | 1696 | 237 | 1.78E-30 | 0.139740566 | #N/A |
| predicted RNA | - | 266 | 37 | 8.02E-10 | 0.139097744 | #N/A |
| LMRG_01779 | APA family basic amino acid/polyamine antiporter | 72 | 10 | 3.67E-23 | 0.138888889 | lmo2469 |
| LMRG_00760 | hypothetical protein | 36 | 5 | 1.02E-13 | 0.138888889 | lmo1310 |
| LMRG_01376 | N-acetyl-gamma-glutamyl-phosphate reductase | 58 | 8 | 2.16E-18 | 0.137931034 | lmo1591 |
| LMRG_00980 | dihydroorotate oxidase | 51 | 7 | 5.29E-14 | 0.137254902 | lmo1833 |
| LMRG_00400 | flagellar basal-body rod protein FlgC | 633 | 85 | 8.02E-41 | 0.134281201 | lmo0711 |
| LMRG_05035 | Leu tRNA | 1337 | 178 | 1.08E-27 | 0.133133882 | #N/A |
| predicted RNA | - | 370 | 49 | 3.61E-11 | 0.132432432 | #N/A |
| LMRG_00778 | tRNA pseudouridine synthase B | 152 | 20 | 1.08E-32 | 0.131578947 | lmo1328 |
| LMRG_00399 | flagellar basal-body rod protein FlgB | 604 | 79 | 1.27E-41 | 0.130794702 | lmo0710 |
| predicted RNA | antisense: LMRG_01743 | 177 | 23 | 1.17E-10 | 0.129943503 | #N/A |
| predicted RNA | - | 524 | 68 | 5.21E-20 | 0.129770992 | #N/A |
| LMRG_02012 | nitrite transporter NirC | 509 | 66 | 3.81E-33 | 0.129666012 | lmo0912 |
| predicted RNA | - | 324 | 42 | 2.16E-18 | 0.12962963 | #N/A |
| LMRG_01584 | hypothetical protein | 124 | 16 | 2.06E-16 | 0.129032258 | #N/A |
| LMRG_00397 | flagellar biosynthesis protein fliS | 1993 | 257 | 5.01E-35 | 0.12895133 | lmo0708 |
| LMRG_00406 | hypothetical protein | 1247 | 160 | 1.60E-36 | 0.128307939 | lmo0717 |
| predicted RNA | - | 219 | 28 | 1.83E-13 | 0.127853881 | #N/A |
| LMRG_01241 | argininosuccinate synthase | 90 | 11 | 5.43E-32 | 0.122222222 | lmo2090 |
| LMRG_00405 | flagellar protein export ATPase FliI | 550 | 66 | 1.79E-41 | 0.12 | lmo0716 |
| LMRG_00985 | aspartate carbamoyltransferase | 34 | 4 | 3.73E-18 | 0.117647059 | lmo1838 |
| predicted RNA | - | 1199 | 134 | 3.87E-22 | 0.1117598 | #N/A |
| LMRG_00841 | simple sugar transport system ATP-binding protein | 127 | 14 | 1.26E-55 | 0.11023622 | lmo1389 |
| LMRG_00978 | orotate phosphoribosyltransferase | 37 | 4 | 3.01E-15 | 0.108108108 | lmo1831 |
| predicted RNA | - | 192 | 20 | 8.11E-19 | 0.104166667 | #N/A |
| predicted RNA | - | 279 | 29 | 5.63E-31 | 0.103942652 | #N/A |
| LMRG_00842 | simple sugar transport system permease | 165 | 17 | 5.55E-56 | 0.103030303 | lmo1390 |
| LMRG_01579 | aspartate aminotransferase | 235 | 24 | 1.18E-75 | 0.10212766 | lmo2252 |
| LMRG_00403 | flagellar motor switch protein FliG | 592 | 60 | 8.87E-68 | 0.101351351 | lmo0714 |
| LMRG_01976 | acetyl-CoA synthetase | 773 | 78 | 8.55E-63 | 0.100905563 | lmo2720 |
| LMRG_00858 | formate acetyltransferase | 355 | 35 | 9.14E-72 | 0.098591549 | lmo1406 |
| LMRG_00984 | dihydroorotase | 64 | 6 | 1.11E-35 | 0.09375 | lmo1837 |
| LMRG_00376 | hypothetical protein | 270 | 25 | 2.17E-74 | 0.092592593 | lmo0688 |
| LMRG_00366 | flagellar biosynthesis protein FliR | 119 | 11 | 1.32E-41 | 0.092436975 | lmo0678 |
| LMRG_00387 | hypothetical protein | 338 | 31 | 2.43E-45 | 0.091715976 | lmo0698 |
| predicted RNA | - | 1163 | 105 | 1.17E-62 | 0.090283749 | #N/A |
| LMRG_00706 | hypothetical protein | 1530 | 137 | 4.97E-90 | 0.089542484 | lmo1257 |
| LMRG_00396 | flagellar hook-associated protein 2 | 1259 | 111 | 3.48E-82 | 0.08816521 | lmo0707 |
| LMRG_00401 | flagellar hook-basal body complex protein FliE | 337 | 29 | 6.46E-57 | 0.086053412 | lmo0712 |
| LMRG_00368 | flagellar biosynthesis protein FlhA | 95 | 8 | 1.26E-74 | 0.084210526 | lmo0680 |
| LMRG_02347 | mannose-specific PTS system IID component | 216 | 18 | 2.87E-83 | 0.083333333 | lmo0098 |
| LMRG_00364 | flagellar biosynthetic protein FliP | 410 | 34 | 6.85E-123 | 0.082926829 | lmo0676 |
| LMRG_05037 | Thr tRNA | 401 | 33 | 1.30E-37 | 0.082294264 | #N/A |
| predicted RNA | - | 379 | 30 | 2.01E-36 | 0.079155673 | #N/A |
| LMRG_00982 | carbamoyl-phosphate synthase large subunit | 89 | 7 | 2.07E-117 | 0.078651685 | lmo1835 |
| LMRG_02908 | hypothetical protein | 140 | 11 | 1.14E-38 | 0.078571429 | #N/A |
| LMRG_00367 | flagellar biosynthetic protein FlhB | 272 | 21 | 5.76E-133 | 0.077205882 | lmo0679 |
| LMRG_01064 | formate acetyltransferase | 689 | 53 | 4.57E-119 | 0.076923077 | lmo1917 |
| predicted RNA | - | 281 | 21 | 3.94E-48 | 0.074733096 | #N/A |
| LMRG_01580 | polar amino acid transport system ATP-binding protein | 436 | 32 | 5.02E-157 | 0.073394495 | lmo2251 |
| LMRG_01838 | hypothetical protein | 151 | 11 | 1.30E-66 | 0.072847682 | lmo2410 |
| LMRG_00979 | orotidine 5'-phosphate decarboxylase | 127 | 9 | 3.19E-70 | 0.070866142 | lmo1832 |
| LMRG_01269 | hypothetical protein | 466 | 33 | 3.13E-154 | 0.070815451 | lmo2115 |
| predicted RNA | - | 505 | 35 | 3.98E-56 | 0.069306931 | #N/A |
| LMRG_00983 | carbamoyl-phosphate synthase small subunit | 130 | 9 | 8.52E-85 | 0.069230769 | lmo1836 |
| predicted RNA | - | 188 | 13 | 2.11E-45 | 0.069148936 | #N/A |
| predicted RNA | - | 772 | 52 | 5.12E-116 | 0.067357513 | #N/A |
| LMRG_02874 | flagellar motor switch protein FliN | 611 | 40 | 2.69E-133 | 0.065466448 | lmo0675 |
| LMRG_00374 | chemotaxis protein MotB | 872 | 54 | 7.09E-193 | 0.061926606 | lmo0686 |
| predicted RNA | - | 326 | 20 | 3.99E-66 | 0.061349693 | #N/A |
| LMRG_00748 | HTH-type transcriptional regulator glnR | 87 | 5 | 1.99E-60 | 0.057471264 | lmo1298 |
| LMRG_01268 | hypothetical protein | 301 | 17 | 1.10E-188 | 0.056478405 | lmo2114 |
| predicted RNA | - | 341 | 19 | 2.61E-95 | 0.055718475 | #N/A |
| LMRG_00369 | flagellar biosynthesis regulator FlhF | 222 | 12 | 2.50E-219 | 0.054054054 | lmo0681 |
| predicted RNA | - | 674 | 36 | 5.75E-87 | 0.053412463 | #N/A |
| LMRG_00375 | hypothetical protein | 512 | 26 | 5.22E-242 | 0.05078125 | lmo0687 |
| predicted RNA | antisense: LMRG_01332 | 355 | 17 | 1.73E-116 | 0.047887324 | #N/A |
| predicted RNA | - | 315 | 15 | 1.95E-118 | 0.047619048 | #N/A |
| LMRG_02345 | mannose-specific PTS system IIAB component manL | 939 | 42 | 0 | 0.044728435 | lmo0096 |
| predicted RNA | - | 249 | 11 | 1.37E-135 | 0.044176707 | #N/A |
| LMRG_00046 | fumarate reductase flavoprotein subunit | 570 | 25 | 0 | 0.043859649 | lmo0355 |
| LMRG_05003 | Thr tRNA | 412 | 18 | 1.15E-143 | 0.04368932 | #N/A |
| predicted RNA | - | 208 | 9 | 1.68E-121 | 0.043269231 | #N/A |
| LMRG_00373 | chemotaxis protein MotA | 849 | 36 | 0 | 0.042402827 | lmo0685 |
| predicted RNA | - | 386 | 15 | 7.22E-180 | 0.038860104 | #N/A |
| predicted RNA | antisense: LMRG_01332 | 284 | 11 | 5.53E-170 | 0.038732394 | #N/A |
| LMRG_02346 | mannose-specific PTS system IIC component | 549 | 20 | 0 | 0.036429872 | lmo0097 |
| LMRG_00371 | chemotaxis protein methyltransferase | 191 | 6 | 0 | 0.031413613 | lmo0683 |
| LMRG_00986 | uracil permease | 535 | 16 | 0 | 0.029906542 | lmo1839 |
| predicted RNA | - | 373 | 11 | 0 | 0.029490617 | #N/A |
| predicted RNA | - | 606 | 13 | 0 | 0.021452145 | #N/A |
| LMRG_01332 | bifunctional acetaldehyde-CoA/alcohol dehydrogenase | 4772 | 98 | 0 | 0.020536463 | lmo1634 |
| predicted RNA | - | 3702 | 61 | 0 | 0.01647758 | #N/A |
| LMRG_00372 | hypothetical protein | 1276 | 21 | 0 | 0.01645768 | lmo0684 |
| LMRG_01479 | glutamate decarboxylase | 809 | 12 | 0 | 0.014833127 | lmo2363 |
| predicted RNA | - | 237 | 3 | 0 | 0.012658228 | #N/A |
| predicted RNA | - | 660 | 8 | 0 | 0.012121212 | #N/A |
| LMRG_01480 | glutamate/gamma-aminobutyrate antiporter | 1094 | 12 | 0 | 0.010968921 | lmo2362 |
